# UPLC-MS/MS analysis and bioactivity comparison of wild and cultivated *Taihangia rupestris* leaves: antioxidant and α-glucosidase inhibitory activities with active compound screening

**DOI:** 10.1039/d6ra00256k

**Published:** 2026-05-26

**Authors:** Jin-tuo Yin, De-mao Wang, Xue-chun Wu, Juan Lu, Zheng-ming Qian, De-qiang Li, Rui Feng

**Affiliations:** a Department of Pharmacy, The Fourth Hospital of Hebei Medical University Shijiazhuang 050011 Hebei Province China fengrui-125@163.com; b Department of Pharmacy, The Second Hospital of Hebei Medical University No. 215, Heping West Road Shijiazhuang 050000 Hebei Province China lideqiang@hebmu.edu.cn; c College of Medical Imaging Laboratory and Rehabilitation, Xiangnan University Chenzhou Hunan Province 423000 China; d Key Laboratory of State Administration of Traditional Chinese Medicine, Shenzhen HEC Industrial Development Co., Ltd Shenzhen Guangdong Province 518053 China

## Abstract

*Taihangia rupestris* Yu & Li (*T. rupestris*) was a nationally protected plant and its active compounds exhibit antidiabetic potential, but systematic phytochemical and pharmacological investigations remain limited. To balance conservation and utilization, the chemical composition and bioactivities (antioxidant and α-glucosidase inhibitory effects) of wild and cultivated *T. rupestris* were compared, followed by active compound screening. UPLC-MS/MS was used to identify chemical constituents in wild, mountain-cultivated, and foothill-cultivated samples, with multivariate analysis for differential components. The TFC and TPC, and the antioxidant capacity were evaluated *via* FRAP, CUPRAC, TRC, and DPPH assays. Additionally, α-glucosidase inhibition was assessed *via* IC_50_ determination. Online HPLC-ABTS and ultrafiltration-LC/MS (UF-LC/MS) were employed to screen antioxidants and α-glucosidase inhibitors (α-GIs), respectively, with molecular docking validating binding mechanisms. As a result, among 114 identified compounds, 111 showed significant environment-dependent variations, primarily flavonoids, phenolics, and terpenoids. Foothill-cultivated plants exhibited upregulated flavonoids/phenolics (*e.g.*, rutin and gallic acid derivatives, *P* < 0.05) and superior antioxidant activity (Trolox equivalents: FRAP 367.18 ± 1.03; CUPRAC 572.40 ± 0.82) and α-glucosidase inhibition (IC_50_ 0.2775 µg mL^−1^) *versus* wild (IC_50_ 0.4948 µg mL^−1^) and mountain-cultivated samples (IC_50_ 0.5425 µg mL^−1^). Ten antioxidants were screened, with seven also acting as α-GIs. UF-LC/MS and docking confirmed 8 α-GIs (binding energy < −5 kcal mol^−1^), where phenolic hydroxyl groups formed hydrogen bonds with ASP residues of α-glucosidase. Cultivated *T. rupestris* (especially foothill-grown) outperformed wild plants in bioactives composition and efficacy, serving as a sustainable alternative. Flavonoids and phenolics contributed to its antidiabetic potential *via* dual antioxidant and enzyme-inhibitory effects, supporting further pharmaceutical development.

## Abstract 2

*Taihangia rupestris* Yu & Li (*T. rupestris*) was a nationally protected plant and its active compounds exhibit antidiabetic potential, but systematic phytochemical and pharmacological investigations remain limited.

## Graphical abstract

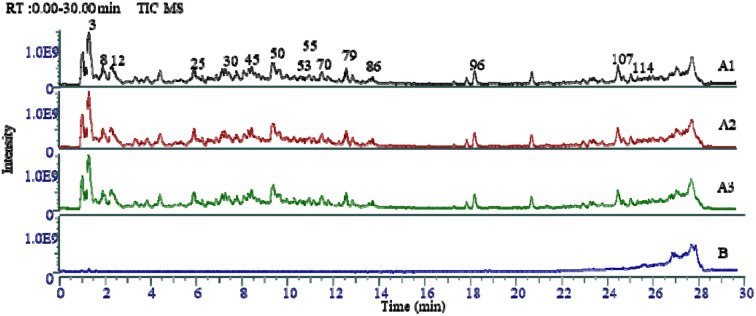

## 1 Introduction

Diabetes mellitus (DM), a chronic and incurable disease with escalating global prevalence, significantly impairs patients' quality of life due to its associated complications.^1^ Current pharmacological interventions, primarily relying on Western medicines, often cause intolerable adverse effects like abdominal distension, hypoglycemia, and diarrhea with long-term use.^2^ Furthermore, managing complications frequently necessitates polypharmacy, inadvertently increasing the risk of adverse drug reactions and further diminishing quality of life. Consequently, developing novel therapeutics with fewer side effects and potential multi-target efficacy remains a paramount research objective.

Reactive oxygen species (ROS) are closely implicated in diabetic complications such as peripheral neuropathy, diabetic foot, retinopathy, and nephropathy.^3^ Additionally, α-glucosidase inhibitors (α-GIs), which mitigate postprandial hyperglycemia by suppressing polysaccharide hydrolysis, show therapeutic potential not only for diabetes but also for conditions sharing pathological similarities with its complications (*e.g.*, cardiovascular/cerebrovascular diseases, neuritis).^4^ Therefore, screening for antioxidants and α-GIs represents a promising strategy for identifying natural products with medicinal potential against diabetes and its complications.

*Taihangia rupestris* Yu & Li (*T. rupestris*), a nationally protected Class II plant, has been traditionally used in folk medicine to treat tinea and as an adjunct therapy for diabetes.^5^ While five chemical constituents (β-sitosterol, ursolic acid, 2α,3β-dihydroxyursolic acid, gallic acid and sericoside) have been identified, possessing anti-inflammatory, antidiabetic, antitumor, and neurotrophic properties,^6^ the plant's complex composition likely harbors additional bioactive components effective against diabetes and its complications. However, wild *T. Rupestris* is scarce and endangered, raising a critical question: Can artificially cultivated plants match or surpass the bioactivity of wild specimens to enable sustainable utilization? Currently, research is limited to preliminary chemical characterization, lacking systematic comparisons of bioactivity, pharmacological mechanisms, or composition between wild and cultivated variants under different ecological conditions.

To address these gaps, firstly, the chemical profiles and relative abundances of constituents in wild *T. rupestris* with those cultivated under distinct ecological conditions (mountain *vs.* foothill) were compared using high-resolution liquid chromatography-tandem mass spectrometry (UPLC-MS/MS). Then, their antioxidant potential relevant to oxidative imbalance was evaluated through quantifying total flavonoid/polyphenol content and multiple *in vitro* assays (ferric reducing antioxidant power, cupric ion reducing capacity, total reducing power, DPPH radical scavenging). Besides, their α-glucosidase inhibitory activity was also assessed using *p*-NPG as substrate. Lastly, rapidly screening for specific antioxidant compounds and α-GI ligands within the complex extract was conducted using our established techniques of online extraction HPLC-ABTS (OLE-HPLC-ABTS) and affinity ultrafiltration coupled with HPLC-MS/MS, respectively.^7–9^ Molecular docking further analyzed binding modes of identified enzyme inhibitors. By integrating comparative phytochemistry with bioactivity assessments and rapid screening, this work seeks to determine the viability of cultivated *T. rupestris* as a substitute for the wild plant and identify key bioactive constituents responsible for its antioxidant and antidiabetic potential.

## 2 Materials and methods

### 2.1 Materials

#### 2.1.1 Chemicals and reagents

The standards of rutin (purity ≥98%), gallic acid (purity ≥98%), chlorogenic acid (purity 99.54%) and quinodimethacrylate (purity ≥98%) were respectively provided by the China National Institute for Food and Drug Control (Beijing, China), Beijing Solebao Technology Co., Ltd (Beijing, China), Shanghai Ronghe Pharmaceutical Science and Technology Development Co., Ltd (Shanghai, China), and Shanghai Aladdin Biochemical Technology Co., Ltd (Shanghai, China). α-glucosidase (EC 3.2.1.20, 32.4 U mg^−1^), and p-nitrophenyl β-d-glucopyranoside (pNPG) were provided by Shanghai Baumann Bio-technology Co., Ltd (Shanghai, China). 2,2-diphenyl-1-picrylhydrazyl (DPPH) was purchased from ApexBio Biotechnology Co., Ltd (USA). HPLC-grade methanol, ethanol and acetonitrile were purchased from Merck KGaA (Germany). Formic acid (analytical grade) was bought from Thermo Fisher Scientific Shanghai Co., Ltd (Shanghai, China). Watson's distilled water and ultrapure water were supplied by Milli-Q Pure Water Manufacturing System (Millipore, USA).

#### 2.1.2 Plant materials

The dried leaves of wild *T. rupestris* (ys), foothill-cultivated *T. rupestris* (sx), and mountain-cultivated *T. rupestris* (ss) were collected in January 2020. They were provided by Yu-lian Yan from Yan Ohdao Village, Live Water Town, Wu'an County, Hebei Province, and identified by Dr Yongli Liu, an expert in traditional Chinese medicine from the Food and Drugs and Medical Devices Inspection and Research Center of Hebei Province, as *T. rupestris* of the rosaceae family. The remaining medicinal materials were kept at the Hebei Department of Pharmacy, the Second Hospital of Medical University, Shijiazhuang, Hebei Province.

Detailed environmental parameters for each sampling site were recorded. Ys were collected from the native habitat at an altitude of 1200–1350 m (mountain brown soil, low organic matter; mean annual temperature 8.5 °C, range −15 °C to 28 °C; mean annual precipitation 560 mm; high UV radiation, 6–8 h direct sunlight). Ss were grown at 800–950 m on the same mountain range (amended loam soil; mean annual temperature 10.2 °C, range −10 °C to 30 °C; precipitation 580 mm; moderate light, 4–6 h direct sunlight; occasional manual watering). Sx were grown at 150–250 m in the foothills (fertile alluvial soil; mean annual temperature 13.5 °C, range −5 °C to 36 °C; precipitation 620 mm; full sunlight, 8–10 h; regular manual irrigation). These parameters were obtained from local meteorological station records and soil analyses performed at the Hebei Provincial Institute of Agricultural Environment during the collection period (January 2020).

#### 2.1.3 Instruments

High performance liquid chromatography (HPLC) was supplied by Waters Corporation (USA), consisting of controller, on-line degasser, binary pump, autosampler, column oven. Orbitrap Exploris 120 Mass Spectrometer, TraceFounder 5.1 and FreeStyle™ 1.8 were provided by Thermo Fisher Scientific (USA).

Agilent Model 1260 HPLC with on-line degasser, quaternary pump, column oven, autosampler, and DAD detector and Agilent Model 6530 Q-TOF-MS mass spectrometer were provided by Agilent Technologies Ltd (USA). Molecular Operating Environment (MOE) software 2015.10 was provided by Chemical Computing, Canada Group Inc. (CA). ChemDraw 20.0 was provided by PerkinElmer Instruments, Inc. (USA).

### 2.2 Preparation of extracts

The samples were crushed and mixed with an appropriate amount of 50% methanol at a ratio of 100 mg mL^−1^ to soak the dried herb powder for 30 min at ambient temperature. Subsequently, they were ultrasonicated for 30 min. After cooling down to room temperature, the samples were made up for any weight loss and centrifuged at 15 000 rpm for 10 min at 4 °C. The supernatant was then retained and stored at 4 °C for future use.

### 2.3 Phytochemical analysis of the extracts by UPLC-MS/MS

LC conditions: the American Waters Atlantis T3 column (150 × 2.1 mm, 3 µm) was used for analysis. Column temperature was maintained at 25 °C, and sample tray temperature was at 6 °C. The mobile phase consisted of water containing 0.05% formic acid (A) and a mixed solvent of methanol and acetonitrile (1 : 1) containing 0.05% formic acid (B). Flow rate was 0.3 mL min^−1^. Gradient elution was adopted with the following program settings: 0–0.5 min, 10% B; 0.5–7.0 min, 10–30% B; 7.0–8.0 min, 30–36% B; 8.0–18.0 min, 36–65% B; 18.0–25.0 min, 65–95% B; 25.0–26.0 min, 95% B; 26.0–26.1 min, 95–10% B. The chromatographic column was equilibrated with 10% B for 4 minutes. The total elution time was 30 min, and the injection volume was 1 µL.

MS condition: electro spray ion (ESI) source was utilized for analysis. In negative ion mode detection, source jet voltage was set as −2500 V; in positive ion mode detection, source jet voltage was set as 3400 V. The source temperature was kept at 350 °C, ion transport tube temperature was 320 °C; Sheath gas, aux gas and sweep gas was 10 arb, 7 arb and 1 arb, respectively. Ion scanning range was 100–1300 *m*/*z*. The polarity of output voltage was that both positive and negative are swept in the same direction.

### 2.4 The TFC and TPC, antioxidant capacity and α-glucosidase inhibition *in vitro*

The antioxidant potential of extracts was evaluated through quantifying total flavonoid/polyphenol content and multiple *in vitro* assays (ferric reducing antioxidant power (FRAP), cupric ion reducing antioxidant capacity (CUPRAC), total reducing capacity (TRC), and 2,2-diphenyl-1-picrylhydrazyl (DPPH) radical scavenging activity) as previous reports with simplified modifications.^10–15^ As well, α-glucosidase inhibition was assayed as previously described. For detailed experimental procedures, please refer to the SI.

### 2.5 Discovery of antioxidants and α-glucosidase inhibitors

#### 2.5.1 Off-line extraction of samples

About 0.2 g of sx powder sample was taken, weighed precisely, and put in a 15 mL centrifuge tube. Methanol (50%, 4 mL) was added to make a mixture of 0.05 g mL^−1^. The supernatant was taken after ultrasonic (power 380 W, frequency 37 kHz) assisted extraction for 20 min, then through the 0.22 µm organic filter membrane, the renewed filtrate was sealed at 4 °C for future use.

The ultrasonication power (380 W) and the same batch of sx plant material was used as in Section 2.2.

#### 2.5.2 Characterization of the compounds in sx extract

The compounds in the extract of sx were characterized by HPLC-Q-TOF-MS. HPLC separations were carried out on an Agilent Poroshell 120 EC-C18 chromatographic column at 40 °C. Gradient elution was performed using water containing 0.1% (v/v) formic acid (A)-acetonitrile (B) as mobile phases. The flow rate was processed at 1 mL min^−1^ as follows: 0–0.01 min, 10% B; 0.01–5 min, 12% B; 5–20 min, 18% B; 20–25 min, 30% B; and 25–26 min, 42%B. The detection wavelength was at 254 nm. The injection volume was 2 µL.

MS conditions were as follows: ESI electrospray ionization source, dry gas (N_2_) flow rate of 8 L min^−1^, dry gas temperature of 350 °C, nebulization pressure of 35 psig, capillary voltage of 3500 V, scanning mode of positive/negative ion mode, collision-induced dissociation voltage of 120 V, and ion scanning range of *m*/*z* 50–1000.

#### 2.5.3 Antioxidants screening by OLE-HPLC-ABTS

##### 2.5.3.1 Preparation of standards solution

Appropriate amounts of the control of gallic acid, chlorogenic acid and rutin were taken, dissolved in 50% methanol, prepared into a mixed solution at a concentration of 90 µg mL^−1^ of gallic acid, 70 µg mL^−1^ of chlorogenic acid and 180 µg mL^−1^ of rutin, and passed through a 0.22 µm organic filter membrane.

##### 2.5.3.2 Preparation of ABTS solution

Appropriate amounts of ABTS and potassium persulfate were weighed and dissolved in water to prepare an aqueous solution containing 3.5 mmol L^−1^ of ABTS and 2.5 mmol L^−1^ of potassium persulfate at a final concentration, and the reaction was carried out at 4 °C, protected from light, for 12 h. The reaction was diluted with anhydrous ethanol, and the absorbance of the ABTS solution was adjusted to be 1.0 at 750 nm on the ultraviolet spectrophotometer.^16^

##### 2.5.3.3 Online analysis by OLE-HPLC-ABTS

About 2 mg of the mixture of the sx powder and diatomaceous earth (1 : 20) were weighed, the mixture was filled into a clean blank precolumn, sealed at both ends with a filter membrane, and mounted into a matching precolumn sleeve to form an on-line extraction cell for the samples. The prepared on-line sample extraction cell and the blank pre-column were connected to the liquid chromatography system through a six-way valve, and the on-line extraction was carried out with the mobile phase.

HPLC separations were carried out as description in Section 2.5.2. The separated compounds mixed with ABTS solution (0.4 mL min^−1^) pumped by an external liquid phase pump, and reacted in 2 m × 0.25 µm PEEK tubes before entering a UV detector for the detection of antioxidant active ingredients at 750 nm.

#### 2.5.4 α-glucosidase inhibitors screening

##### 2.5.4.1 Ultrafiltration procedure

Experimental group: *T. rupestris* test solution (200 µL) was mixed with α-glucosidase (6 U mL^−1^, 200 µL). After incubation at 37 °C for 1 h, 200 µL of the mixed solution was added to a 3 KD ultrafiltration centrifuge tube and centrifuged at 10 000 g for 30 min to isolate compounds that were not bound to the enzyme. The unbound compounds were washed by adding 100 µL of 5% methanol PBS solution, and then centrifuged again (10 000 g, 30 min) and repeated three times.

Control group: α-glucosidase solution (6 U mL^−1^) was heated in boiling water for 30 min to obtain inactivated enzyme solution. The inactivated enzyme solution was used to replace the active α-glucosidase solution, and the rest of the operation was the same as the experimental group.

Finally, the centrifuged solutions of control and experimental groups were analyzed by HPLC. The binding degree of each component was calculated according to equation:
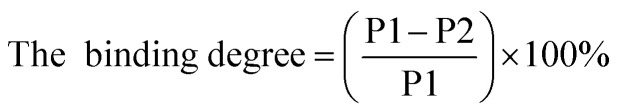
(P1 and P2 are the peak areas of the components in the control group (inactivated enzyme group) and in the experimental group (interaction with active enzyme), respectively.)

##### 2.5.4.2 Molecular docking

Molecular docking was performed with MOE software, and protein molecules related to α-glucosidase with X-ray diffraction crystal information and carrying ligands were downloaded from the website RCSB PDB (https://www.rcsb.org/). The 2D structure of the compounds to be screened were downloaded in the PubChem website (https://pubchem.ncbi.nlm.nih.gov/). For docking, the stationary macromolecular receptor was selected, the docking pocket was the location of the ligand-carrying site, and the ligand small molecules were allowed to change their location and structural configuration for semi-flexible docking, with London dG = 60 and the number of exported conformations (GBVI/WSA dG) = 30. The optimal docking conformation was selected to be the one that had the highest number of interacting bonds with the protein receptor, and at the same time, had the lowest docking energy.

### 2.6 Statistical analysis

Below was a concise description of the statistical methods employed and the specific contexts in which they were applied.

Principal component analysis (PCA) was performed on the normalized peak areas of all identified compounds to visualize global differences in chemical composition among ys, ss, and sx samples. The Shapiro–Wilk test was used to assess the normality of the peak area data for each compound. Since most data deviated significantly from normality (*P* < 0.05), non-parametric tests were subsequently applied. Specifically, pairwise comparisons of compound abundances between sample groups (ys *vs.* ss, ys *vs.* sx, and ss *vs.* sx) were conducted using the Mann–Whitney *U* test (or the Kruskal–Wallis test for multi-group comparisons). Statistical significance was defined as *P* < 0.05. For the bioactivity assays – including TFC, TPC, FRAP, CUPRAC, TRC, DPPH radical scavenging activity, and α-glucosidase inhibition (IC_50_) – group comparisons were performed using one-way ANOVA followed by Tukey's post hoc test when normality (Shapiro–Wilk) and homogeneity of variances (Levene's test) assumptions were met. Otherwise, the Kruskal–Wallis test was used as a non-parametric alternative.

All statistical analyses were carried out using SPSS 25.0 and GraphPad Prism 8.0. A *P*-value threshold of 0.05 was considered statistically significant for all tests.

## 3 Results

### 3.1 Qualitative identification analysis results and method reproducibility validation results

**Fig. 1:**
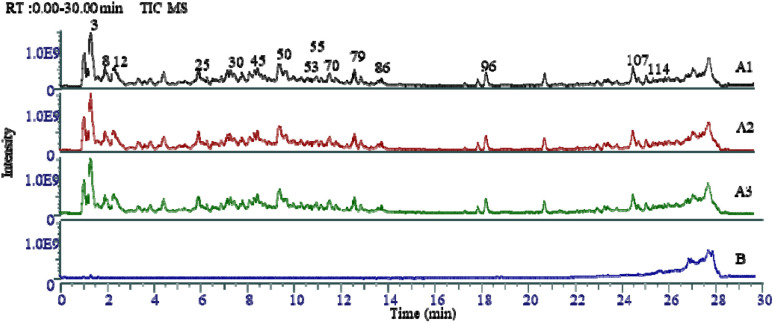
TIC of *T. rupestris* mixed sample (A1, A2 and A3: TIC of mixed samples for repeatability; B: TIC of extracted solvent for blank control).

The results of the characterization of the compounds in the extracts of the mixed *T. rupestris* samples were shown in STable 1. A total of 114 compounds were screened out as the possible components in the mixed extracts of the *T. rupestris*. The total ion current diagram (TIC) of the mixed samples of the *T. rupestris* was shown in Fig. 1. The results of the repeatability validation were shown in Fig. 1A1–A3.

### 3.2 Comparative analysis of components in *T. rupestris* from different growth environments

#### 3.2.1 PCA of chemical constituents among different growth environments

The peak areas corresponding to the compounds were compared after the three batches of samples and the mixed samples were analyzed with the same LC-MS conditions and qualitative analytical methods, respectively.

**Fig. 2:**
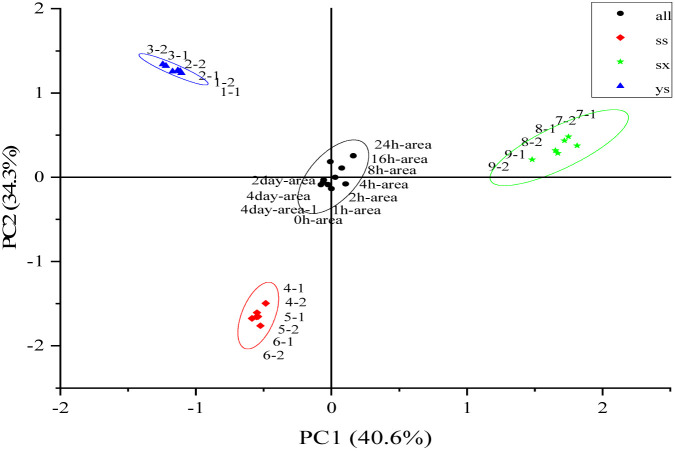
PCA plots of the constituents of *T. rupestris* extracts from different growth environments.

After the peak area data was standardized, a straight line through the data center point was identified to maximize the variance of the data projected onto the line with the shortest projection distance after standardization, which was the first principal component PC1 (40.6%), and the straight line perpendicular to the principal component PC1 over the data center point of the sample was the second principal component PC2 (34.3%), with a cumulative contribution of the two principal components of 74.9%. The new values corresponding to the sample data projected on the plane shared by principal components PC1 and PC2 were taken as the coordinates under the new coordinate system, and the PCA score map was obtained (see Fig. 2).

In Fig. 2, ys (1-1, 1-2, 2-1, 2-2, 3-1, 3-2), ss (4-1, 4-2, 5-1, 5-2, 6-1, 6-2), sx (7-1, 7-2, 8-1, 8-2, 9-1, 9-2) were the results of principal component analysis of the same growth environment for each of the three different samples of ys, ss, and sx with six times of repeated testing. The results showed that the spacing between the results of six times of repeated testing of the same samples was small and within the confidence circle, representing that the difference in principal components between the same samples after repeated testing was not significant; the spacing between the different samples was larger, representing that there was a significant difference between the principal components contained in the different samples. All (0 h-area, 1 h-area, 2 h-area, 4 h-area, 8 h-area, 16 h-area, 24 h-area, 2 day-area, 4 day-area, 4 day-area-1) represented the results of 10 repetitions of the test after the equal mixing of the three different samples, and the 10 test results were spaced at a close distance, which were all within the circle of confidence and there was no intersection with the three different samples, which indicated that there were significant differences between the principal components of the mixed sample and the principal components of all three different samples.

#### 3.2.2 Comparative analysis of compound composition: Venn diagram, heatmap, and differential expression

**Fig. 3:**
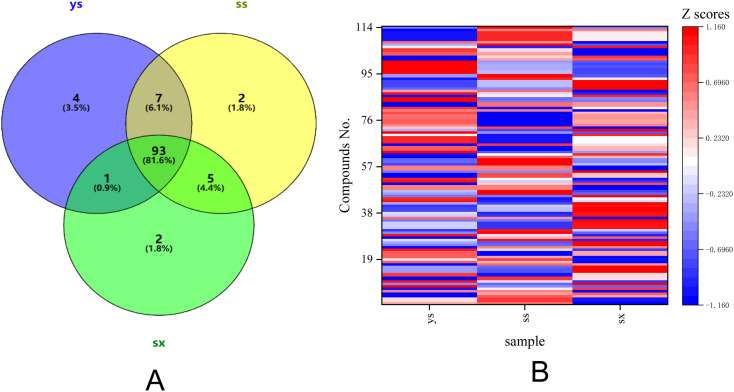
Differential chemical profiles of *T. rupestris* across growth environments. (A) Venn diagram of shared and unique compounds. (B) Heatmap of compound abundance (red: up-regulation; blue: down-regulation).

Differential compositional profiles of *T. rupestris* across different growth environments were presented in Fig. 3 and SI Table 2. The results of the Venn plot (Fig. 3A) analysis showed that four compounds that appeared only in ys were isobutyl 4-hydroxybenzoate (43), kaempferol-7-*O*-beta-d-glucopyranoside (74), oroxin A (85), and (+)-nootkatone (112); two compounds appearing only in ss were scoparone (59) and germacrone (109); and two compounds appearing only in sx were 2′-*o*-galloylhyperin (72) and salicylic acid (82). There were five compounds missing (ss ∩ sx ≠ ys) in ys only in the three different batches of *T. rupestris* samples, namely l-tryptophan (24), caffeic acid (50), isoferulic acid (64), pinoresinol 4-o-glucoside (66) and astringin (93); one (ys ∩ sx ≠ ss) compound missing in ss only was geniposide (41); and seven (ys ∩ ss ≠ sx) compounds missing in sx only were raffinose (1), helicid (19), p-hydroxy-cinnamic acid (36), androsin (46), chrysin (98), 7,8-dihydroxyflavone (99), ursolic acid (106).

**Fig. 4:**
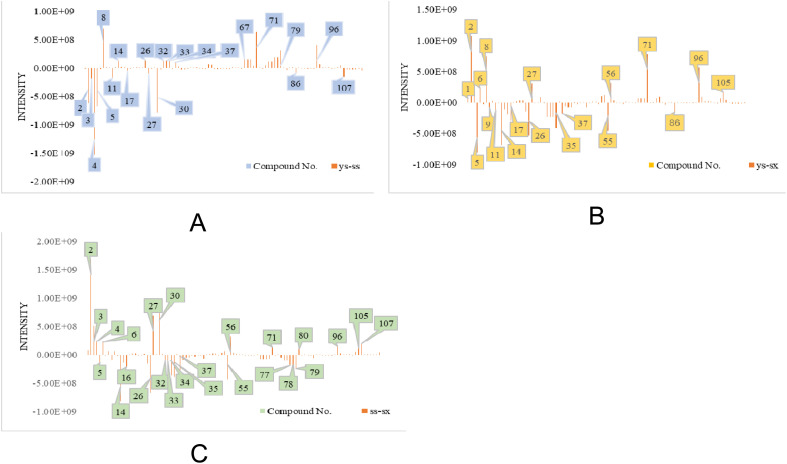
Comparative bar chart of composition differences in *T. rupestris* (A: ys-ss, B: ys-sx, C: ss-sx).

In addition, there were 93 compounds shared by *T. rupestris* grown in three different environments. According to the Shapiro Wilk test (S–W), the peak area data corresponding to the same compound in the same sample extract showed a non-normal distribution (*P* < 0.05). Non-parametric testing was used to test the significant differences in peak area data corresponding to the same compound in different samples (see STable 2 for details). Among them, compared with ss, 46 compounds were upregulated and 39 compounds were downregulated in ys with significant differences (*P* < 0.05); compared with sx, there were 41 compounds upregulated and 37 compounds downregulated in ys with significant differences (*P* < 0.05); compared with sx, there were 41 compounds upregulated and 44 compounds downregulated in ss with significant differences (*P* < 0.05). The median peak areas of the compounds with significant differences among different samples were normalized and a heatmap was plotted in Fig. 3B, with red representing up-regulation, and the darker the color, the more obvious up-regulation; and blue representing down-regulation, and the darker the color, the more obvious down-regulation. The difference in peak areas of the compounds with significant differences in different *T. rupestris* samples was represented by a bar graph (Fig. 4).

#### 3.2.3 Comparison of TFC, TPC, antioxidant and α-glucosidase inhibitory power *in vitro*

**Fig. 5:**
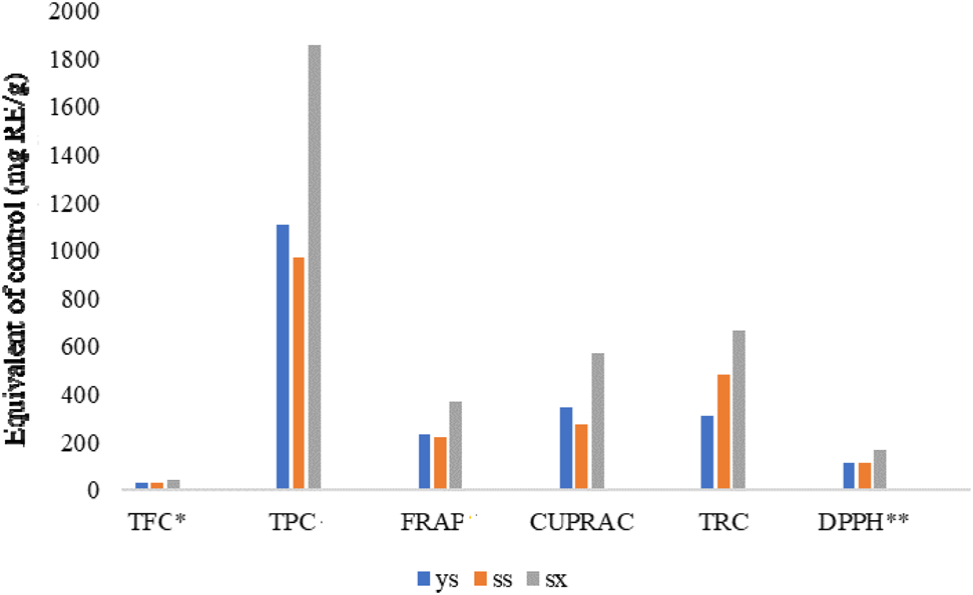
Comparison of antioxidant capacity of *T. rupestris* in different growth environments.

**Fig. 6:**
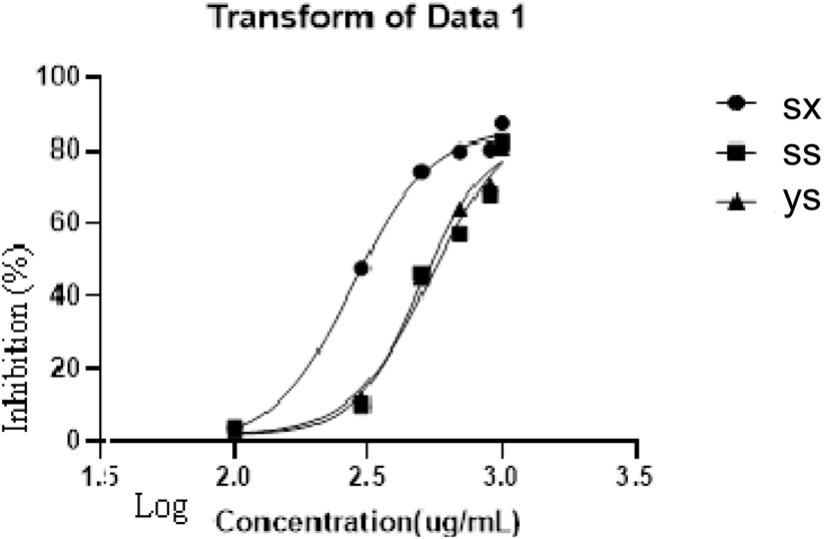
α-glucosidase inhibition of different *T. rupestris*.

TFC, TPC and antioxidant capacities of *T. rupestris* in different growth environments were shown in Fig. 5 and Table 1. The higher the equivalent of the control substance, the stronger the content and antioxidant capacity. The sx had a higher equivalent than ys and ss, so the sx sample had the highest TFC, TPC and the strongest antioxidant capacity measured under experimental conditions. Fig. 6 showed the results of α-glucosidase inhibition assay. The IC_50_ values of sx, ys and ss were 0.2775 µg mL^−1^, 0.4948 µ g mL^−1^, and 0.5425 µg mL^−1^, respectively. Sx had the strongest enzyme inhibition, followed by ys and ss. With strongest antioxidant and α-glucosidase inhibitory activity, sx sample was selected for the followed study of active compounds discovery.

**Table 1:** Comparison of TFC, TPC and antioxidant capacity of *T. rupestris* in different growth environments

Antioxidant testing method	Control substance	Equivalent of the control substance ± RSD% (mg RE per g)			Standard curve
		ys	ss	sx	
TFC^a^	Rutin	28.67 ± 0.87	29.24 ± 2.23	43.07 ± 2.04^c^	*y* = 2.777*x* − 0.002, *R*^2^ = 0.999
TPC^a^	Gallic acid	1104.55 ± 1.11	968.62 ± 0.82	1859.39 ± 0.62^c^	*y* = 6.571*x* − 0.023, *R*^2^ = 0.997
FRAP^b^	Trolox	234.83 ± 1.64	219.53 ± 0.88^d^	367.18 ± 1.03^c^	*y* = 2.364*x* + 0.016, *R*^2^ = 1.000
CUPRAC^b^	Trolox	342.35 ± 1.18	272.94 ± 1.60^d^	572.40 ± 0.82^c^	*y* = 0.187*x* − 0.003, *R*^2^ = 0.999
TRC^b^	Trolox	310.03 ± 2.57	479.35 ± 1.78^d^	667.62 ± 1.56^c^	*y* = 0.361*x* + 0.043, *R*^2^ = 0.980
DPPH^b^	Trolox	112.61 ± 2.71	112.28 ± 1.88	168.15 ± 2.19^c^	*y* = 76.433*x* − 2.014, *R*^2^ = 0.995

a Statistical evaluation was performed by Kruskal–Wallis test.

b Statistical evaluation was performed by ANOVA test. Different letters indicate difference in the tested extracts (*p* < 0.05).

c sx compared with ys or ss.

d ss compared with ys.

### 3.3 Discovery of antioxidants and α-glucosidase inhibitors in sx sample

#### 3.3.1 Qualitative analysis of the sx sample

**Fig. 7:**
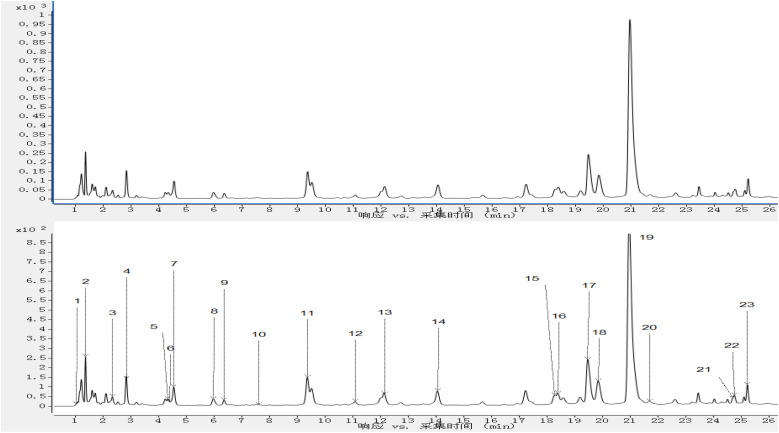
Chromatogram of off-line extraction of *T. rupestris*.

The results of the compositional analysis of sx sample were shown in Table 2. A total of 23 compounds were identified through literature and offline database comparison, combined with offline extraction chromatograms (Fig. 7), which showed high matches and good peak shapes with the relevant secondary ion fragments and basic physicochemical properties in the database. The 23 compounds listed in Table 2 were a subset of the 114 compounds identified in the pooled sample (STable 1), specifically those that were present in the sx extract and could be confidently detected under the HPLC-Q-TOF-MS conditions used for activity screening.”

**Table 2:** Offline extraction of mass spectrum data of chemical constituents from *T. rupestris*

No.	Name	RT (min)	Molecular formula	Accurate molecular weight	Parent ion type	Parent ion	Fragmentation ion
1	Proline	1.179	C_5_H_9_NO_2_	115.0633	[M + H]^+^	116.0707	**70.0651**
2	Hexahydroxydiphenoyl-d-glucose	1.625	C_20_H_18_O_14_	482.06965	[M − H]^−^	481.0628	**300.998**, 275.0198, 249.0411, 229.0122
3	Phenylalanine	2.445	C_9_H_11_NO_2_	165.079	[M + H]^+^	166.0859	**120.0759**, 103.0541, 77.0379
4	Casuariin	3.143	C_34_H_24_O_22_	784.07592	[M − H]^−^	783.0681	**300.9985**, 275.0197
5	Epicatechin	4.497	C_15_H_14_O_6_	290.079	[M + H]^+^	291.0855	291.0867, **139.0350**
6	Tryptophan	4.525	C_11_H_12_N_2_O_2_	204.0899	[M + H]^+^	205.0967	**188.0699**, 146.0591
7	Pedunculagin	4.818	C_34_H_24_O_22_	784.07592	[M − H]^−^	783.0681	**300.9991**, 275.0199
8	Chlorogenic acid	6.189	C_16_H_18_O_9_	354.0951	[M + H]^+^	355.1017	**163.0377**, 145.0222, 117.0269
9	Catechin	6.501	C_15_H_14_O_6_	290.079	[M + H]^+^	291.0855	291.0867, **139.0350**
10	BLicoagroside B	7.756	C_18_H_24_O_12_	432.1268	[M + H]^+^	433.1326	**127.0388**, 145.0478
11	Luteolin 7-glucuronosyl-(1,2)-glucuronide	9.568	C_27_H_26_O_18_	638.1119	[M + H]^+^	639.1176	**463.0860**, 287.0542
12	Tricetin 3′-methyl ether 7,5′-diglucuronide	11.284	C_28_H_28_O_19_	668.1225	[M + H]^+^	669.1283	**439.969**, 317.0648
13	Ellagic acid glucoside	12.384	C_20_H_16_O_13_	464.0591	[M + H]^+^	465.0631	465.0648, **303.0147**
14	Procyanidin B2 3′-*O*-gallate	14.196	C_37_H_30_O_16_	730.1534	[M + H]^+^	731.1615	411.1092, 303.0507, 153.0174
15	4′-*O*-Arabinofuranosylellagic acid	18.561	C_19_H_14_O_12_	434.0485	[M + H]^+^	433.0383	433.0374, **300.9952**
16	Ellagic acid 2-rhamnoside	18.690	C_20_H_16_O_12_	448.0642	[M − H]^−^	447.0528	447.0528, **300.9959**
17	Ellagic acid 4-*O*-xylopyranoside	19.809	C_19_H_14_O_12_	434.0485	[M − H]^−^	433.0383	433.0372, 300.9950, 299.9876
18	Ellagic acid	20.198	C_14_H_6_O_8_	302.0062	[M − H]^−^	300.996	299.9876, 288.9934, 245.0050, 145.0267
19	Potentillin	21.229	C_41_H_28_O_26_	936.087	[M − H]^−^	935.0748	767.0673, 300.9961
20	Isoquercetin	21.932	C_21_H_20_O_12_	464.0955	[M + H]^+^	465.1033	303.0489
21	Luteolin-7-*O*-glucoside	24.975	C_21_H_20_O_11_	448.1006	[M + H]^−^	449.1057	287.054
22	Kaempferol-3-*O*-glucuronoside	25.052	C_21_H_18_O_12_	462.0798	[M + H]^−^	463.0870	287.0545
23	Ducheside A	25.518	C_20_H_16_O_12_	448.0641	[M − H]^−^	447.0546	315.0126, 299.9886

#### 3.3.2 Online antioxidants screening

##### 3.3.2.1 Efficiency evaluation of online sample extraction

The efficiency results of online sample extraction were shown in SFig. 1. When the sample was extracted online for the second time without peaks, indicating the high efficiency of the method for the first extraction online.

##### 3.3.2.2 Repeatability examination

The repeatability test results were shown in SFig. 2. The chromatograms of repeated injections could overlap, indicating good repeatability.

#### 3.3.3 Online antioxidants screening

**Fig. 8:**
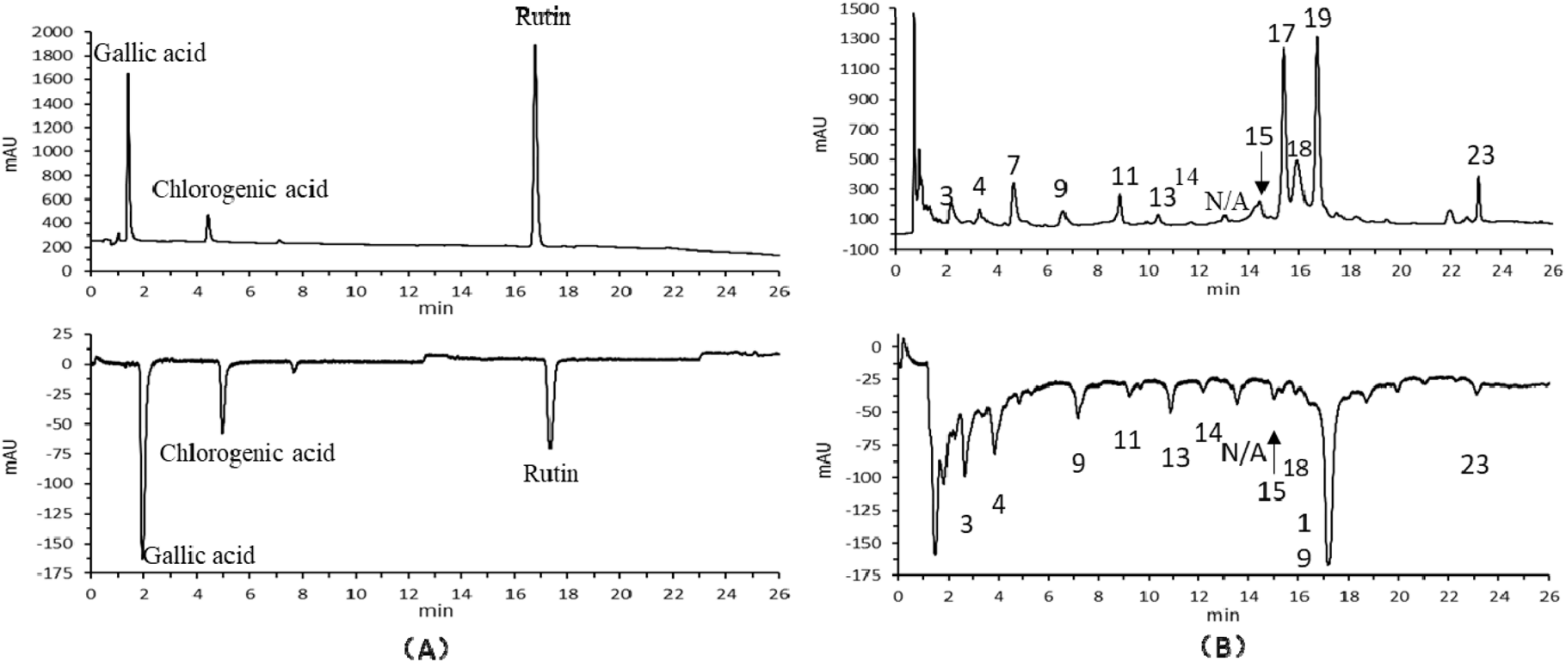
Chromatograms of control substances (A) and *T. rupestris* (B) for on-line antioxidant analysis.

The results of online antioxidant (OLE-HPLC-ABTS) analysis were shown in Fig. 8. Combined with the analysis in Table 2, the corresponding absorption peaks of the control gallic acid, chlorogenic acid, and rutin showed inverted peaks after elimination of ABTS, and after analyzing the samples online in the same way, it could be seen that there were 3 (phenylalanine), 4 (casuariin), 9 (catechin), 11 (luteolin 7-glucuronosyl-(1,2)-glucuronide), 13 (ellagic acid glucoside), 14 (procyanidin B2 3′-*o*-gallate), N/A (unknown), 15 (4′-*o*-arabinofuranosylellagic acid), 18 (ellagic acid), 19 (potentillin), 23 (ducheside A) absorption peaks showing inverted peaks, proving that the compounds corresponding to these peaks could eliminate ABTS and had antioxidant effects.

#### 3.3.4 α-glucosidase inhibitors screening by affinity ultrafiltration

The results of α-GI screening were shown in SFig. 3 and Table 3. According to the chromatogram of the filtrate after ultrafiltration (SFig. 3), there were eight compounds that could bind to the active α-glucosidase in *T. rupestris*, and the one with the highest binding rate was the procyanidin B2 3′-*O*-gallate with a binding rate of 47.76% (Table 2).

**Table 3:** Results of ultrafiltration centrifugation of *T. rupestris*

No.	Chemical compounds	Retention time (min)	Binding rate %
1	Phenylalanine	2.338	21.56
2	Casuariin	3.552	20.00
3	Ellagic acid glucoside	11.007	39.18
4	Procyanidin B2-3′-*O*-gallate	12.270	47.76
5	4′-*O*-Arabinofuranosylellagic acid	15.022	15.21
6	Ellagic acid 4-*O*-xylopyranoside	16.174	10.56
7	Ellagic acid	16.549	27.46
8	Potentillin	17.339	29.97

#### 3.3.5 Optimal interaction conformation for molecular docking

The optimal interaction conformation for molecular docking results were shown in SFig. 4, and the docking results were detailed in STable 3. The docking energies, in ascending order, were (a) potentillin (−8.0015 kcal mol^−1^), (b) ellagic acid 4-*o*-xylopyranoside (−7.6251 kcal mol^−1^), (c) ellagic acid glucoside (−7.3979 kcal mol^−1^), (d) procyanidin B2-3′-*o*-gallate (−6.9372 kcal mol^−1^), (e) 4′-*o*-arabinofuranosylellagic acid (−6.6741 kcal mol^−1^), (f) casuariin (−6.0355 kcal mol^−1^), (g) ellagic acid (−5.3858 kcal mol^−1^), (h) phenylalanine (−5.0462 kcal mol^−1^). The smaller the docking energy, the easier it was for the compound to bind to the protein molecule.

The binding bonds between the above components and 2QMJ protein were mainly hydrogen bonds, which were reversibly connected to ASP amino acid residues in the protein through oxygen atoms by hydroxyl, ketone, aldehyde, or carboxylic acid groups of the compound. For example, the optimal interaction conformations of (a), (c), (d), (e), and (f) was connected to ASP542 amino acid residue, the optimal interaction conformations of (a), (c), and (d) were connected to ASP203 amino acid residue, and (c) and (e) were connected to ASP327 amino acid residue through hydrogen bonding.

## 4 Discussion

In this study, the chemical composition analysis and bioactivity assessment of different samples (ys, ss, sx) of *T. rupestris* revealed the material basis and mechanism of action of its antioxidant and α-glucosidase inhibitory activities, and explored its medicinal potential. The following was a comprehensive discussion of the chemical composition differences, bioactivity correlation and molecular mechanism.

### 4.1 Association of chemical composition differences with antioxidant activity

Recent metabolomics studies have demonstrated that UPLC-MS/MS-based profiling combined with chemometrics can effectively discriminate wild from cultivated medicinal plants and identify environment-sensitive bioactive metabolites.^17,18^ Applying this approach to *T. rupestris*, we identified 114 compounds, of which 111 exhibited significant abundance variations among ys, ss, and sx leaves (STable 2). The differentially accumulated metabolites were predominantly phenolics, flavonoids, and terpenoids. The net change in known antioxidant compounds (upregulated minus downregulated) was highest in sx, followed by ys then ss – a ranking that precisely matched TFC, TPC, and the *in vitro* antioxidant activity measured by FRAP, CUPRAC, TRC, and DPPH assays (Table 1 and Fig. 5). Hence, the overall antioxidant capacity arises from the cumulative effect of multiple compounds, not any single component.

#### 4.1.1 Mechanisms of key antioxidant compounds

Several differentially accumulated compounds are well-characterized antioxidants. Gallic acid and chlorogenic acid – significantly higher in sx than in ys or ss – bear multiple phenolic hydroxyl groups that act as potent hydrogen atom donors, directly accounting for their radical scavenging activity.^14^ Ellagic acid derivatives contain catechol moieties enabling sequential radical quenching *via* hydrogen atom transfer (HAT) and proton-loss electron-transfer (PLET), with the ability to neutralize up to two radicals per cycle. Corilagin boosts cellular antioxidant capacity by activating the Nrf2/HO-1 pathway, while procyanidin B1 and epicatechin enhance systemic antioxidant defenses through Nrf2-mediated upregulation of detoxifying enzymes.^19^ Their lower abundance in ys and ss partially explains the reduced antioxidant performance of these samples.

#### 4.1.2 Environmental regulation of flavonoid and phenolic biosynthesis

Flavonoids are synthesized *via* the phenylpropanoid pathway. The entry enzyme phenylalanine ammonia-lyase (PAL) is rate-limiting, and the committed step is catalyzed by chalcone synthase (CHS).^20,21^ Downstream enzymes (CHI, F3H, FLS) produce flavonols, flavones, and proanthocyanidins. Phenolic acids originate from the shikimate pathway.

The three growth sites differ in abiotic factors that regulate these pathways. Light and UV radiation upregulate PAL, CHS, and FLS expression *via* photoreceptors and UVR8.^22^ Here, sx plants received the longest daily sunlight (8–10 h) and had the highest flavonoid levels. Temperature also modulates activity: moderate warmth (mean 13.5 °C) enhances PAL activity, whereas low temperatures at the high-altitude wild site (down to −15 °C) may suppress enzyme function.^23^ Soil fertility further contributes: fertile alluvial soil at the sx site provides abundant carbon skeletons for secondary metabolism.^24^

We therefore propose that the upregulated flavonoid and phenolic levels in sx are primarily driven by enhanced light exposure and favorable temperature – factors that upregulate key biosynthetic genes rather than by classic abiotic stress (which is more prominent at high altitudes). Thus, foothill cultivation creates an environment that promotes antioxidant accumulation, offering a sustainable alternative to wild harvesting.

### 4.2 The material basis of α-glucosidase inhibitory activity

Among the constituents of *T. rupestris* with antidiabetic effect or α-glucosidase inhibition were quinic acid (5),^25^ gallic acid (14),^26^ epicatechin (30),^19^l-leucine (10),^27^ geraniin (25),^28^ geniposide (41),^29^ brevifolincarboxylic acid (45),^30^ emodin (48),^31^ isoferulic acid (64),^32^ quercetin (68),^33,34^ oroxin A (85),^35^ salicylic acid (82)^5^ and β-sitosterol.^5,36^ Their distribution varied: (1) sx: 6 compounds were upregulated (*e.g.*, quinic acid, gallic acid, geraniin, geniposide, including 2 components with obvious upregulation). (2) ys: 3 compounds were upregulated (*e.g.*, oroxin A, quercetin, including 1 component with the same content as ss). (3) ss: 3 compounds were upregulated (*e.g.*, quinic acid, epicatechin, emodin, 1 component with the same content as ys, and 1 component with obvious up regulation). Given the broader and more pronounced upregulation in sx, its α-glucosidase inhibitory potential was highest, consistent with bioassay results. Notably, flavonoid efficacy in α-glucosidase inhibition might relate to C3' substituents on the B ring.^33,34^

### 4.3 Additional pharmacological activities

In addition to the above-mentioned antidiabetic or α-glucosidase inhibitory constituents, there were also constituents possessing a variety of pharmacological effects such as anti-inflammatory, antibacterial, and anticancer effects in *T. rupestris*, and most of these constituents possessed a variety of different pharmacological effects at the same time. Kaempferol-7-o-β-*o*-glucopyranoside (74), a constituent identified only in ys samples, had anti-inflammatory effects,^37^ and oroxin A (85) had anti-cancer and anti-inflammatory effects,^38^*etc.* (+)-Nootkatone (112) has antiplatelet,^39^ anti-inflammatory effects.^40^ Only identified in ss samples of the compound scoparone (59) had a reduction of pulmonary fibrosis^41^ and inhibition of pancreatic cancer, *etc*.^42^ Germacrone (109) had an inhibition of cancer cell proliferation.^43^ Components identified only in sx samples were 2′-*o*-galloylhyperin (72) with anti-inflammatory effects,^44,45^ and salicylic acid (82) with anticancer,^46^ anti-inflammatory and skin softening keratin. Pyrogallol (15), a non-specific constituent of *T. rupestris* identified in three samples from three different growth environments, had anti-inflammatory, anti-malarial and other pharmacological effects,^46^ and so on. Diabetic patients were often accompanied by complications such as poor coagulation, neuritis, peripheral nerve atrophy and limb necrosis due to poor peripheral circulation, *etc.* A variety of constituents in *T. rupestris*, such as geniposide (41), in addition to its therapeutic effect on diabetes, also had a neuroprotective effect,^38^ and these effects could alleviate and treat diabetes and its complications to a certain extent.

### 4.4 Molecular basis of α-glucosidase inhibition

Ultrafiltration assay showed 8 compounds that could bind to α-glucosidase, namely phenylalanine, casuariin, ellagic acid glucoside, procyanidin B2-3′-*o*-gallate, 4′-*o*-arabinofuranosylellagic acid, ellagic acid, potentillin and ellagic acid 4-*o*-xylopyranoside.^9^ A cross-reference between these eight binders and the global metabolomics dataset (STables 1 and 2) was provided in STable 4. Among them, only phenylalanine was consistently detected in the global UPLC-MS/MS profiling; the other seven binders were not annotated in the global dataset due to their low abundance or poor ionization under the original Orbitrap conditions. Nevertheless, they were successfully identified in the second, more sensitive analysis performed on the same batch of sx material, confirming that all eight binders originate from the same chemical matrix. Online antioxidant analysis showed that all compounds bound to the enzyme exhibited antioxidant activity except ellagic acid-4-*o*-xylopyranoside. The antioxidant power of *T. rupestris* was positively correlated with the α-glucosidase inhibition. This phenomenon might be due to the involvement of groups with antioxidant capacity in the enzyme-compound binding site.^15^

Molecular docking revealed: (1) binding mechanism: phenolic hydroxyl and carboxyl groups bind to enzyme active sites (*e.g.*, amino acid residues) *via* hydrogen bonding. (2) Constitutive relationship: the difference between snake tannins (binding energy −8.0015 kcal mol^−1^) and proanthocyanidins B2-3′-*o*-gallate (binding rate 47.76%) suggested the influence of spatial site resistance and electronic effects on binding affinity; (3) energy *vs.* affinity: Potentillin had the lowest binding energy (−8.0015 kcal mol^−1^) but lower observed binding (29.97%), while procyanidin B2-3′-*o*-gallate showed higher binding (47.76%) despite moderate energy (−6.9372 kcal mol^−1^), suggesting conformational fit influences affinity. (4) Redox regulation hypothesis: the antioxidant moiety might enhance the inhibitory effect by protecting the enzyme active center or modulating its conformation.

*T. rupestris* exhibited robust bioactivity, with sx displaying superior antioxidant and antidiabetic potential due to enriched flavonoid/phenolic compounds. Its pharmacological profile parallels or surpasses wild-type samples (ys/ss), supporting its use as a medicinal alternative. The correlation between antioxidant capacity and α-glucosidase inhibition might stem from shared binding motifs (*e.g.*, phenolic hydroxyls) in enzyme-compound interactions. Further studies should explore structure–activity relationships and *in vivo* efficacy.

### 4.5 OLE-HPLC-ABTS analysis for rapid antioxidant screening

Building on the identification of differential compounds and their antioxidant activities, we further employed online HPLC-ABTS (OLE-HPLC-ABTS) to directly visualize which individual components in the complex extract are responsible for radical scavenging. This system integrates pressurized liquid micro-extraction, HPLC separation, and post-column ABTS radical cation reaction, enabling real-time screening without offline fractionation.^7,8^ As shown in Fig. 8, inverted peaks indicate positive antioxidant activity. Among the 23 identified compounds in the foothill-cultivated (sx) extract, 11 exhibited such activity: phenylalanine, casuariin, catechin, luteolin 7-glucuronosyl-(1,2)-glucuronide, ellagic acid glucoside, procyanidin B2-3′-*O*-gallate, an unknown compound, 4′-*O*-arabinofuranosylellagic acid, ellagic acid, potentillin, and ducheside A. Notably, all but one (ellagic acid 4-*O*-xylopyranoside) also bound to α-glucosidase in the ultrafiltration assay (Table 3), revealing a strong functional overlap between antioxidant capacity and α-glucosidase inhibition. This overlap is structurally plausible: phenolic hydroxyl and carboxyl groups enable both electron transfer (radical scavenging) and hydrogen bonding with ASP residues in the α-glucosidase active site, as confirmed by molecular docking. Such dual functionality is particularly relevant for diabetes management, where oxidative stress and postprandial hyperglycemia are intertwined. Although the OLE-HPLC-ABTS method is primarily qualitative, it serves as an efficient initial screen to pinpoint candidate antioxidants from complex mixtures before quantitative dose–response validation.

## 5 Conclusion

This study analyzed the phytochemical composition and bioactivities of *T. rupestris* from different growth environments. HPLC-MS/MS identified 114 compounds, primarily flavonoids and phenolics, with the highest levels in low-altitude cultivated samples, correlating with stronger antioxidant and α-glucosidase inhibitory (α-GI) effects. Online HPLC-ABTS screening and ultrafiltration revealed that most antioxidant components also inhibited α-glucosidase. Molecular docking suggested hydrogen bonding between phenolic hydroxyl groups and enzyme ASP residues as the key mechanism. The findings propose cultivated *T. rupestris* as a viable wild substitute, with dual-active phenolics supporting its potential in diabetes management. This work provides a foundation for further antidiabetic drug development from this plant.

## Author contributions

Jin-tuo Yin: conceptualization, methodology, investigation, writing – original draft. De-mao Wang, writing – original draft. Xue-chun Wu: formal analysis, investigation, validation. Juan Lu: formal analysis, investigation. Zheng-ming Qian: methodology, investigation. De-qiang Li: supervision, project administration, funding acquisition.

## Conflicts of interest

There are no conflicts to declare.

## Supplementary Material

RA-016-D6RA00256K-s001

## Data Availability

Data are available upon reasonable request. Supplementary information (SI) is available. See DOI: https://doi.org/10.1039/d6ra00256k.
