# Supplementary material for: UPLC-MS/MS analysis and bioactivity comparison of wild and cultivated Taihangia rupestris leaves: antioxidant and α-glucosidase inhibitory activities with active compound screening
Source: RSC Adv. 2026 May 26;16(31):28230–42. doi: 10.1039/d6ra00256k (PMC13213544; doi:10.1039/d6ra00256k)
Supplement: RA-016-D6RA00256K-s001 [file RA-016-D6RA00256K-s001.pdf]

Supplementary Information (SI) for RSC Advances.
This journal is © The Royal Society of Chemistry 2026

UPLC-MS/MS Analysis and Bioactivity Comparison of Wild and Cultivated *Taihangia rupestris* Leaves: Antioxidant and α-Glucosidase Inhibitory Activities with Active Compound Screening

Jin-tuo Yin[1#], De-mao Wang[2#], Xue-chun Wu[2], Juan Lu[2], Zheng-ming Qian[3,4], De-qiang Li[2*], Rui Feng[1*]

1. Department of Pharmacy, the Fourth Hospital of Hebei Medical University, Shijiazhuang 050011, Hebei Province, China

2. Department of Pharmacy, the Second Hospital of Hebei Medical University, Shijiazhuang 050000, Hebei province, China

3. College of Medical Imaging Laboratory and Rehabilitation, Xiangnan University, Chenzhou, Hunan province 423000, China

4. Key Laboratory of State Administration of Traditional Chinese Medicine, Shenzhen HEC Industrial Development Co., LTD., Shenzhen, Guangdong province 518053, China

* Corresponding authors:

Deqiang Li, E-mail: lideqiang@hebme.edu.cn
Rui Feng, E-mail: fengrui-125@163.com

[#] The two authors contributed equally to this work.

1 *The TFC and TPC, antioxidant capacity and α-glucosidase inhibition in vitro*

1.1 Determination of total flavonoid content (TFC)

The most commonly used method for the determination of flavonoid content, the $Al(NO_3)_3$-$NaNO_2$-NaOH colorimetric method, was selected to determine the total flavonoid content [10]. 0.1 mL of the sample extract was precisely measured in a 10 mL measuring flask, $NaNO_2$ solution (0.05 g/mL, 0.5 mL), $Al(NO_3)_3$ solution (0.1 g/mL, 0.5 mL), and NaOH solution (4%, 5 mL) were added in sequence, and then, the volume was set with 85% methanol. After the reaction was completed and centrifuged (10000 rpm, 3 min), 0.1 mL of the supernatant was taken in a 96-well enzyme plate, the absorbance was measured at 510 nm, and the average value was taken after several parallel measurements.

The sample extraction solution was replaced with 85% methanol solution as a blank control. The sample extraction solution was replaced with 0.5, 1.0, 1.5, 2.0, 2.5, and 3.0 mL of rutin the control solutions (0.1 mg/mL), and then, processed according to the above method to detect the absorbance of the control solutions at 510 nm wavelength. Finally, a straight line was fitted with the measured results using the concentration of rutin (mg/mL) as the horizontal coordinate, and the average absorbance after deducting the blank (A) as the vertical coordinate, which was used as the TFC standard curve. The rutin equivalents of the sample powder TFC were calculated according to equation (1):

$$equivalent\ of\ the\ control\ product\ (mg\ RE/g) = \frac{x \times dilution\ times \times V}{M} \quad (1)$$

(x: the amount equivalent to the control contained in the measured solution calculated from the corresponding standard curve, mg/mL; dilution: the ratio of the sample extract to the concentration at the time of measurement; V: volume of extraction solvent used in the sample extract, mL; M: the weight of precisely weighed *T. rupestris* powder, g)

1.2 Determination of total phenolic content (TPC)

In view of the limitations of the method for the determination of total flavonoid

content [11], the forintol method was chosen for the determination of total phenol content as a supplement to the determination of antioxidant compounds content. The sample extract (100 mg/mL) was diluted 10-fold with 85% methanol and 0.1 mL was placed in a 10 mL measuring flask. Forintol reagent (0.5 mL), purified water and of Na_2CO_3 solution (10%, 1 mL) were added into the measuring flask. 0.2 mL of the reacted solution was taken into a 96-well enzyme labeling plate, the absorbance was determined at 765 nm, and take the average value was taken after at least three parallel measurements.

The sample extract was replaced with the extraction solvent as a blank control. The sample extract was replaced with 0.25, 0.50, 0.75, 1.00, 1.25, 1.50, and 1.75 mL of 0.1 mg/mL gallic acid control solution, according to the above method, the absorbance value of the control products at 765 nm was measured, and a straight line was fitted by taking the gallic acid concentration (mg/mL) as the horizontal coordinate, and the average absorbance after deducting the blank (A) as the vertical coordinate. A straight line was fitted as the TPC standard curve, and the gallic acid equivalent of TPC of the sample powder was calculated according to the formula (1).

1.3 Iron Reduction Antioxidant Capacity (FRAP)

FRAP reaction solution was prepared for later use [10,12]. 0.4 mg/mL of the sample extract and the above FRAP reaction solution were put into a 96-well enzyme plate, mixed well, and then the absorbance was measured at 593 nm. The average value was taken after at least three parallel measurements.

The sample extract was replaced with 85% methanol as a blank control solution. The sample extract was replaced with Trolox solution at different concentrations between 0 and 1.0 mmol/L, and then processed and detected by the above method. Finally, a straight line was fitted to the FRAP standard curve by taking the concentration of the Trolox solution (mmol/L) as the horizontal coordinate and the average absorbance (A) after deducting the

blank as the vertical coordinate. The FRAP Trolox equivalent of the sample powder was calculated according to the formula (1).

1.4 Copper ion reduction method (CUPRAC)

Copper chloride solution (10 mmol/L, 1mL), ammonium acetate solution (pH=7.0, 1 mol/L, 1mL), neocuproine solution (7.5 mmol/L, 1mL) and the sample extract (0.5 mg/mL, 0.1 mL) were mixed well, and left at room temperature for 30 min before measuring the absorbance of the reacted solution at 450 nm. The average value was taken after at least three parallel measurements [13].

The sample extract was replaced with 85% methanol as a blank control solution. The sample extract was replaced with Trolox solution diluted with 85% methanol to different concentrations between 0-1.2 mmol/L and processed for detection by the above method. A straight line was fitted to the CUPRAC standard curve by taking the concentration of Trolox solution (mmol/L) as the horizontal coordinate and the average absorbance (A) after deduction of the blank as the vertical coordinate. The Trolox equivalents measured by CUPRAC of the sample powder were calculated according to equation (1).

1.5 Total Reduction Force Method (TRC)

The sample extract (0.25 mg/mL, 0.2 mL), PBS buffer (0.2 mmol/L, 0.5 mL), potassium ferricyanide solution (10 mg/mL, 0.5 mL), trichloroacetic acid (10%, 0.5 mL), purified water (1.7 mL), and ferric chloride solution (0.1%, 0.34 mL) were added sequentially to carry out the reaction. 200 μL of reaction solution was taken in a 96-well enzyme plate to measure the absorbance at 700 nm. The average value was taken after at least three parallel measurements [14].

The sample extract was replaced with 85% methanol as a blank control solution. The sample extract was replaced with Trolox solution diluted with 85% methanol to different concentrations. The concentration of Trolox solution (mmol/L) used for the reaction was

taken as the horizontal coordinate, and the average absorbance (A) after the deduction of the blank was taken as the vertical coordinate, and a straight line was fitted to the TRC standard curve. The TRC Trolox equivalent of the sample powder was calculated according to equation (1).

1.6 DPPH radical scavenging ability

The sample extract (different concentrations, 20 μL) and DPPH solution (0.2 mmol/L, 180 μL) were added into a 96-well enzyme plate, and the absorbance at 517 nm was measured as A1. Methanol (85%, 20 μL) and DPPH solution (0.2 mmol/L, 180 μL) were placed into a 96-well enzyme plate. The absorbance of the solution was measured at 517 nm as A0. A1 and A0 were measured at least three times in parallel and the average value was taken. Different concentrations of Trolox were measured in the same way, and the clearance rate I (%) was calculated according to the clearance rate formula (2). The concentration of Trolox (mmol/L) was used as the horizontal coordinate, and the clearance rate I (%) was used as the vertical coordinate to fit the standard curve of Trolox control, and the IC_{50} values of the sample extract and Trolox were obtained from the standard curve [10].

$$I\,(\%) = \frac{A0 - A1}{A0} \times 100 \qquad (2)$$

1.7 α-Glucosidase Inhibition Assay

The α-glucosidase solution (1 U/mL) and p-NPG solution (2.5 mmol/L) were prepared with PBS, and the sample extract was diluted into several sample dilutions of different concentrations with PBS buffer. P-NPG substrate solution (40 μL) and the solution to be measured (20 μL) were added to the 96-well enzyme labeling plate, mixed with appropriate shaking, and preincubated at 37 °C for 5 min. Then, α-glucosidase (20 μL) was added, and the reaction was incubated at 37 °C for 15 min. Finally, the reaction was terminated by adding Na_2CO_3 (0.1 mol/L, 80 μL) [15].

The absorbance was measured at 405 nm using an enzyme marker, and the absorbance

measured when the solution to be tested was the sample dilution was A0; when the solution to be tested was the sample dilution, and then PBS was substituted for the α-glucosidase assay was A1; when the solution to be tested was the extraction solvent, it was A2; when the solution to be tested was the extraction solvent, and then PBS was substituted for the enzyme solution, it was A3; and the inhibition rate of the reaction was calculated by α-glucosidase (%) = [1 - (A0 - A1)/(A2-A3)]×100, and GraphPad Prism 8 software was used to calculate the IC_{50} value.

STable 1. The compounds of *T. rupestris*

No.	Compound Name	RT (min)	Formula	Adduct	*m/z* (Apex)	*m/z* (Delta (ppm))	Library Score (%)	MS^2 Fragments(*m/z*)
1	Raffinose	1.29	$C_{18}H_{32}O_{16}$	$M+HCO_2$	549.167	-1.118	93	221.06682
2	Sucrose	1.33	$C_{12}H_{22}O_{11}$	M-H	341.108	-1.916	88	89.02438
3	Lactose	1.33	$C_{12}H_{22}O_{11}$	$M+HCO_2$	387.114	-1.654	82	101.02437
4	2-Pyrrolidinecarboxylic acid	1.34	$C_5H_9NO_2$	M+H	116.070	-2.892	86	70.06504
5	Quinic acid	1.41	$C_7H_{12}O_6$	M-H	191.056	-1.056	92	127.03997
6	Maleic acid	1.64	$C_4H_4O_4$	M-H	115.004	-0.753	89	71.01385
7	Nicotinamide	1.94	$C_6H_6N_2O$	M+H	123.055	-1.114	96	96.04425, 80.04945
8	Citric acid	1.95	$C_6H_8O_7$	M-H	191.020	-1.120	93	111.00875, 87.00867
9	L-Tyrosine	1.96	$C_9H_{11}NO_3$	M+H	182.081	-1.052	97	136.07561, 123.04388
10	L-Leucine	1.98	$C_6H_{13}NO_2$	M+H	132.102	-0.667	89	69.06989, 86.09634
11	Adenosine	2.09	$C_{10}H_{13}N_5O_4$	M+H	268.104	-1.122	88	136.06178, 85.02835
12	Guanine	2.25	$C_5H_5N_5O$	M+H	152.057	0.0767	81	110.03486, 135.03003
13	5-Hydroxytryptophan	2.56	$C_{11}H_{12}N_2O_3$	M+H	221.092	-1.304	78	204.0657
14	Gallic acid	3.38	$C_7H_6O_5$	M-H	169.014	-1.164	93	107.01409, 125.02438
15	Pyrogallol	3.38	$C_6H_6O_3$	M-H	125.024	-0.658	81	81.03464, 97.02967
16	5-Hydroxymethylfurfural	3.42	$C_6H_6O_3$	M+H	127.039	-1.013	84	81.03337, 109.02843

No.	Compound Name	RT (min)	Formula	Adduct	*m/z* (Apex)	*m/z* (Delta (ppm))	Library Score (%)	MS^2 Fragments(*m/z*)
17	L-Phenylalanine	3.46	$C_9H_{11}NO_2$	M+H	166.086	-0.747	85	120.08067
18	Cinnamic acid	3.46	$C_9H_8O_2$	M+H	149.060	-0.323	N/A	121.02823
19	Helicid	4.61	$C_{13}H_{16}O_7$	$M+HCO_2$	329.088	-0.682	N/A	108.02161
20	4-Methoxysalicylic acid	4.63	$C_8H_8O_4$	M-H	167.035	-0.863	93	123.04527
21	4-Methyl-6,7-dihydroxycoumarin	4.82	$C_{10}H_8O_4$	M+H	193.049	-0.605	85	147.04443, 103.05424
22	5,7-Dihydroxy-4-methylcoumarin	4.82	$C_{10}H_8O_4$	M+H	193.049	-0.605	85	137.06009, 103.05424
23	3,4-Dihydroxyphenylethanol	4.91	$C_8H_{10}O_3$	M-H	153.056	-0.465	89	123.04504
24	L-Tryptophan	5.72	$C_{11}H_{12}N_2O_2$	M+H	205.097	-1.624	95	118.06494, 146.05992
25	Geraniin	5.99	$C_{41}H_{28}O_{27}$	M-H	951.073	-1.151	87	300.99863
26	Corilagin	6.14	$C_{27}H_{22}O_{18}$	M-H	633.072	-1.504	86	300.99875
27	Procyanidin B1	6.71	$C_{30}H_{26}O_{12}$	M-H	577.135	-1.089	88	407.07733, 289.07272
28	Taxifolin	6.88	$C_{15}H_{12}O_7$	M+H	305.065	-0.893	82	213.05511
29	2-Adamantanone	6.98	$C_{10}H_{14}O$	M+H	151.112	-0.403	71	95.04897
30	Epicatechin	7.49	$C_{15}H_{14}O_6$	M-H	289.071	-1.101	92	245.08253, 109.02943
31	p-Hydroxybenzaldehyde	7.50	$C_7H_6O_2$	M+H	123.044	-1.366	87	95.04914, 67.05434
32	Cryptochlorogenic acid	7.55	$C_{16}H_{18}O_9$	M-H	353.087	-1.414	94	173.04541, 135.04523
33	Chlorogenic acid	7.55	$C_{16}H_{18}O_9$	M-H	353.087	-1.414	94	161.0246, 191.05602

No.	Compound Name	RT (min)	Formula	Adduct	*m/z* (Apex)	*m/z* (Delta (ppm))	Library Score (%)	MS^2 Fragments(*m/z*)
34	1-Caffeoylquinic acid	7.55	$C_{16}H_{18}O_9$	M-H	353.087	-1.414	94	179.03459, 191.05602
35	Methyl gallate	7.55	$C_8H_8O_5$	M-H	183.030	-1.128	65	124.01654, 140.01144
36	p-Hydroxy-cinnamic acid	7.55	$C_9H_8O_3$	M-H	163.040	-0.938	90	119.05014
37	7-Hydroxycoumarin	7.56	$C_9H_6O_3$	M+H	163.039	-1.024	59	107.04874
38	Neochlorogenic acid	7.56	$C_{16}H_{18}O_9$	M+H	355.102	-1.386	93	117.0333, 163.03885
39	Protocatechuic acid	7.81	$C_7H_6O_4$	M-H	153.019	-1.277	90	81.03452, 109.02959
40	Gentisic acid	7.81	$C_7H_6O_4$	M-H	153.019	-1.277	90	109.02959
41	Geniposide	8.00	$C_{17}H_{24}O_{10}$	M-H	387.129	-1.185	58	225.07722
42	Fraxetin	8.26	$C_{10}H_8O_5$	M-H	207.030	-0.923	87	192.0062
43	Isobutyl 4-Hydroxybenzoate	8.32	$C_{11}H_{14}O_3$	M+H	195.101	-0.899	80	95.0489
44	5-Hydroxy-1-tetralone	8.32	$C_{10}H_{10}O_2$	M+H	163.075	-0.542	82	107.049
45	Brevifolincarboxylic acid	8.46	$C_{13}H_8O_8$	M+H	293.029	-1.614	93	219.02873, 191.03387
46	Androsin	8.59	$C_{15}H_{20}O_8$	M-H	327.108	-1.086	85	165.05632
47	Acetophenone	8.95	C_8H_8O	M+H	121.065	-1.837	50	93.06953
48	Emodin	9.03	$C_{15}H_{10}O_5$	M+H	271.060	-1.391	72	173.05992
49	Apigenin	9.03	$C_{15}H_{10}O_5$	M+H	271.060	-1.391	72	197.05972
50	Caffeic acid	9.65	$C_9H_8O_4$	M-H	179.035	-1.317	92	107.0499, 135.04512
51	Asarylaldehyde	9.78	$C_{10}H_{12}O_4$	M+H	197.081	-1.543	78	95.0489

No.	Compound Name	RT (min)	Formula	Adduct	*m/z* (Apex)	*m/z* (Delta (ppm))	Library Score (%)	MS^2 Fragments(*m/z*)
52	Loganin	9.81	$C_{17}H_{26}O_{10}$	M+NH$_4$	408.186	-1.466	N/A	109.06455
53	Taxifolin 7-rhamnoside	10.67	$C_{21}H_{22}O_{11}$	M-H	449.109	-0.776	66	125.02455
54	Astilbin	10.67	$C_{21}H_{22}O_{11}$	M-H	449.109	-0.776	66	151.00357
55	1,2,3,4,6-Pentagalloylglucose	10.83	$C_{41}H_{32}O_{26}$	M-H	939.110	-0.714	77	313.056
56	Naringenin chalcone	10.95	$C_{15}H_{12}O_5$	M+H	273.075	-1.249	94	153.01811
57	Engeletin	10.95	$C_{21}H_{22}O_{10}$	M+H	435.128	-1.051	56	153.01805
58	Fusarenon-X_M+HCOO	10.98	$C_{17}H_{22}O_8$	M+HCOO	399.129	-1.149	N/A	59.01376
59	Scoparone	10.99	$C_{11}H_{10}O_4$	M+H	207.065	-1.422	63	179.07014
60	Citropten	10.99	$C_{11}H_{10}O_4$	M+H	207.065	-1.422	63	179.07014
61	Arglabin	10.99	$C_{15}H_{18}O_3$	M+H	247.132	-1.529	68	187.11096
62	4-Methylumbelliferone	11.01	$C_{10}H_8O_3$	M+H	177.054	-0.911	N/A	149.05954
63	Leucoside	11.38	$C_{26}H_{28}O_{15}$	M-H	579.135	-0.389	87	284.03226
64	Isoferulic acid	11.38	$C_{10}H_{10}O_4$	M-H	193.050	-1.034	92	134.03722
65	Ferulic acid	11.38	$C_{10}H_{10}O_4$	M-H	193.050	-1.034	92	134.03722, 178.02762
66	Pinoresinol 4-O-glucoside	11.40	$C_{26}H_{32}O_{11}$	M-H	519.187	-0.324	85	151.03992
67	Morin	11.60	$C_{15}H_{10}O_7$	M+H	303.050	-1.421	86	229.04921, 137.02299
68	Quercetin	11.60	$C_{15}H_{10}O_7$	M+H	303.050	-1.421	86	153.01823, 165.0179
69	Herbacetin	11.60	$C_{15}H_{10}O_7$	M+H	303.050	-1.421	86	274.04788

No.	Compound Name	RT (min)	Formula	Adduct	*m/z* (Apex)	*m/z* (Delta (ppm))	Library Score (%)	MS^2 Fragments(*m/z*)
70	Isoquercitrin	11.61	$C_{21}H_{20}O_{12}$	M-H	463.088	-1.327	93	300.02731, 271.02463
71	Quercetin 3-O-beta-D-Glucuronide	11.67	$C_{21}H_{18}O_{13}$	M-H	477.067	-1.441	94	151.00371, 301.03503
72	2'-O-Galloylhyperin	11.81	$C_{28}H_{24}O_{16}$	M-H	615.099	-0.162	88	151.0036, 301.03534
73	Nivalenol_M+CH3OO	11.97	$C_{15}H_{20}O_{7}$	M+CH_3OO	359.134	-1.259	N/A	59.01379
74	Kaempferol-7-O-beta-D-glucopyranoside	12.63	$C_{21}H_{20}O_{11}$	M+H	449.107	-1.475	93	287.05502, 153.01836
75	Astragalin	12.64	$C_{21}H_{20}O_{11}$	M-H	447.093	-1.679	90	284.03241, 227.03476
76	Cynaroside	12.64	$C_{21}H_{20}O_{11}$	M-H	447.093	-1.679	90	151.00301, 285.03918
77	Kaempferol	12.65	$C_{15}H_{10}O_{6}$	M+H	287.055	-0.911	88	153.01791, 258.05228
78	Scutellarein	12.65	$C_{15}H_{10}O_{6}$	M+H	287.055	-0.911	88	213.05463
79	Scutellarin	12.74	$C_{21}H_{18}O_{12}$	M-H	461.072	-1.676	91	239.03519, 285.04022
80	Isorhamnetin	12.74	$C_{16}H_{12}O_{7}$	M+H	317.065	-1.533	91	302.04236, 153.01823
81	Fisetin	12.74	$C_{15}H_{10}O_{6}$	M-H	285.040	-1.049	84	135.00845, 229.05051
82	Salicylic acid	13.11	$C_{7}H_{6}O_{3}$	M-H	137.024	-0.711	91	93.03448, 65.03973
83	Azelaic acid	13.23	$C_{9}H_{16}O_{4}$	M-H	187.097	-0.816	92	125.09721, 97.06583
84	Genistin	13.66	$C_{21}H_{20}O_{10}$	M+H	433.112	-1.280	87	271.06015
85	Oroxin A	13.66	$C_{21}H_{20}O_{10}$	M+H	433.112	-1.280	87	271.06015
86	Glabrolide	13.87	$C_{30}H_{44}O_{4}$	M+H	469.330	-1.760	84	95.08536

No.	Compound Name	RT (min)	Formula	Adduct	*m/z* (Apex)	*m/z* (Delta (ppm))	Library Score (%)	MS² Fragments(*m/z*)
87	Phloretin	13.95	$C_{15}H_{14}O_5$	M+H	275.091	-1.442	90	107.04897, 169.04906
88	Linolenic acid ethyl ester	15.52	$C_{20}H_{34}O_2$	M+H	307.263	-1.512	75	81.06974
89	Isopsoralen	15.62	$C_{11}H_6O_3$	M+H	187.039	-1.055	84	131.049
90	Bergapten	16.25	$C_{12}H_8O_4$	M+H	217.049	-1.100	94	202.02591, 202.02591
91	Methoxsalen	16.25	$C_{12}H_8O_4$	M+H	217.049	-1.100	94	202.02591, 189.05432, 185.02281
92	Isobergapten	16.25	$C_{12}H_8O_4$	M+H	217.049	-1.100	94	202.02591, 161.0596
93	Astringin	16.68	$C_{20}H_{22}O_9$	M-H	405.119	-0.979	86	243.06551
94	Narirutin	16.85	$C_{27}H_{32}O_{14}$	M-H	579.172	-0.411	N/A	151.00386
95	Norfenfluramine	18.25	$C_{10}H_{12}F_3N$	M+H	204.099	-2.483	N/A	159.04172
96	Pedunculoside	18.41	$C_{36}H_{58}O_{10}$	M+COOH	695.400	-1.691	82	487.34186
97	Rosamultin	18.41	$C_{36}H_{58}O_{10}$	$M+Cl^-$	685.372	-1.283	83	487.34079
98	Chrysin	20.73	$C_{15}H_{10}O_4$	M-H	253.050	-0.548	86	209.06036, 143.05013
99	7,8-Dihydroxyflavone	20.73	$C_{15}H_{10}O_4$	M-H	253.050	-0.548	86	151
100	Rubiadin	20.73	$C_{15}H_{10}O_4$	M-H	253.050	-0.548	86	181.0661, 209.06036
101	6-Gingerol	21.48	$C_{17}H_{26}O_4$	M-H	293.176	-0.687	87	177.09236, 236.10548
102	6-Shogaol	21.73	$C_{17}H_{24}O_3$	M+H	277.179	-2.240	86	137.05956
103	Medicagenic acid	22.06	$C_{30}H_{46}O_6$	M-H	501.322	-0.946	73	437.30542

No.	Compound Name	RT (min)	Formula	Adduct	*m/z* (Apex)	*m/z* (Delta (ppm))	Library Score (%)	MS² Fragments(*m/z*)
104	Eplerenone	22.82	$C_{24}H_{30}O_6$	M+H	415.211	-1.917	N/A	119.08518
105	Asiatic acid	23.68	$C_{30}H_{48}O_5$	M+H	489.357	-1.055	77	205.15904
106	Ursolic acid	24.19	$C_{30}H_{48}O_3$	M+H	457.367	-0.946	88	95.0853
107	Quillaic acid	24.77	$C_{30}H_{46}O_5$	M-H	485.327	-1.404	86	405.3154, 393.31696
108	Tributyl Phosphate	24.86	$C_{12}H_{27}O_4P$	M+H	267.172	-1.498	N/A	116.99454, 98.98393
109	Germacrone	24.99	$C_{15}H_{22}O$	M+H	219.174	-1.918	76	159.11702, 95.08525
110	Aristolone	24.99	$C_{15}H_{22}O$	M+H	219.174	-1.918	76	121.06452
111	alpha-Cyperone	24.99	$C_{15}H_{22}O$	M+H	219.174	-1.918	76	145.10112
112	(+)-Nootkatone	24.99	$C_{15}H_{22}O$	M+H	219.174	-1.918	76	81.06967
113	Methyl hexadecanoate	25.12	$C_{17}H_{34}O_2$	$M+HCO_2$	315.254	-0.745	87	297.24435, 141.12846
114	alpha-Linolenic acid	25.64	$C_{18}H_{30}O_2$	M+H	279.231	-1.594	86	95.08533, 67.05418

RT: retention time; *m/z*(Apex): mass charge ratio (measured value); MS² Fragments(m/z): secondary ion of mass spectrum.

STable 2. The difference comparison of *T. rupestris* grow in varies environments

No.	Compound Name	Comparison of median peak area					
		ys	ss	sx	ys-ss	ys-sx	ss-sx
1[*]	Raffinose	B	A	-	☆	-	-
2[*]	Sucrose	B	A	C	☆	★	★
3[*]	Lactose	B	A	C	☆	★	★
4[ac]	2-Pyrrolidinecarboxylic acid	B	A	B	☆	=	★
5[*]	Quinic acid	C	B	A	☆	☆	☆
6[bc]	Maleic acid	A	A	B	=	★	★
7[*]	Nicotinamide	C	B	A	☆	☆	☆
8[*]	Citric acid	A	B	C	★	★	★
9[*]	L-Tyrosine	B	C	A	★	☆	☆
10[ab]	L-Leucine	A	B	B	★	★	=
11[*]	Adenosine	C	A	B	☆	☆	★
12	Guanine	A	A	A	=	=	=
13[*]	5-Hydroxytryptophan	A	C	B	★	★	☆
14[*]	Gallic acid	B	C	A	★	☆	☆
15[*]	Pyrogallol	B	C	A	★	☆	☆
16[*]	5-Hydroxymethylfurfural	B	C	A	★	☆	☆
17[ab]	L-Phenylalanine	B	A	A	☆	☆	=
18[bc]	Cinnamic acid	A	A	B	=	★	★
19[*]	Helicid	A	B	-	★	-	-
20[*]	4-Methoxysalicylic acid	A	B	C	★	★	★
21[ac]	4-Methyl-6,7-dihydroxycoumarin	A	B	A	★	=	☆
22[ac]	5,7-Dihydroxy-4-methylcoumarin	A	B	A	★	=	☆
23[*]	3,4-Dihydroxyphenylethanol	A	B	C	★	★	★
24[ab]	L-Tryptophan	-	A	A	-	-	=
25[bc]	Geraniin	B	B	A	=	☆	☆
26[*]	Corilagin	B	C	A	★	☆	☆

No.	Compound Name	Comparison of median peak area					
		ys	ss	sx	ys-ss	ys-sx	ss-sx
27[*]	Procyanidin B1	B	A	C	☆	★	★
28[ab]	Taxifolin	B	A	A	☆	☆	=
29[*]	2-Adamantanone	A	B	C	★	★	★
30[*]	Epicatechin	B	A	C	☆	★	★
31[*]	p-Hydroxybenzaldehyde	C	A	B	☆	☆	★
32[*]	Cryptochlorogenic acid	B	C	A	☆	☆	☆
33[*]	Chlorogenic acid	B	C	A	★	☆	☆
34[*]	1-Caffeoylquinic acid	B	C	A	★	☆	☆
35[*]	Methyl gallate	C	B	A	☆	☆	☆
36[bc]	p-Hydroxy-cinnamic acid	A	A	-	=	-	-
37[*]	7-Hydroxycoumarin	B	C	A	★	☆	☆
38[*]	Neochlorogenic acid	B	C	A	★	☆	☆
39[*]	Protocatechuic acid	C	B	A	☆	☆	☆
40[*]	Gentisic acid	C	B	A	☆	☆	☆
41[*]	Geniposide	B	-	A	-	☆	-
42[*]	Fraxetin	B	C	A	★	☆	☆
43[ab]	Isobutyl 4-Hydroxybenzoate	A	-	-	-	-	-
44[*]	5-Hydroxy-1-tetralone	A	C	B	★	★	☆
45[bc]	Brevifolincarboxylic acid	B	B	A	=	☆	☆
46[*]	Androsin	B	A	-	☆	-	-
47[*]	Acetophenone	B	A	C	☆	★	★
48[bc]	Emodin	A	A	B	=	★	★
49[bc]	Apigenin	A	A	B	=	★	★
50[*]	Caffeic acid	-	B	A	-	-	☆
51[*]	Asarylaldehyde	A	B	C	★	★	★
52[*]	Loganin	A	B	C	★	★	★
53[a]	Taxifolin 7-rhamnoside	B	A	AB	☆	=	=
54[a]	Astilbin	B	A	AB	☆	=	=
55[*]	1,2,3,4,6-Pentagalloylglucose	C	B	A	☆	☆	☆

No.	Compound Name	Comparison of median peak area					
		ys	ss	sx	ys-ss	ys-sx	ss-sx
56[bc]	Naringenin chalcone	A	A	B	=	★	★
57[bc]	Engeletin	A	A	B	=	★	★
58[*]	Fusarenon-X_M+HCOO	C	A	B	☆	☆	★
59[ac]	Scoparone	-	A	-	-	-	-
60[ac]	Citropten	B	A	B	☆	=	★
61[ab]	Arglabin	B	A	A	☆	☆	=
62[*]	4-Methylumbelliferone	C	B	A	☆	☆	☆
63[*]	Leucoside	A	C	B	★	★	☆
64[*]	Isoferulic acid	-	B	A	-	-	☆
65[*]	Ferulic acid	A	C	B	★	★	☆
66[*]	Pinoresinol 4-O-glucoside	-	A	B	-	-	★
67[*]	Morin	A	C	B	★	★	☆
68[*]	Quercetin	A	C	B	★	★	☆
69[*]	Herbacetin	A	C	B	★	★	☆
70[ac]	Isoquercitrin	A	B	A	★	=	☆
71[*]	Quercetin 3-O-beta-D-Glucuronide	A	B	C	★	★	★
72[bc]	2'-O-Galloylhyperin	-	-	A	-	-	-
73[*]	Nivalenol_M+CH3OO	B	A	C	☆	★	★
74[ab]	Kaempferol-7-O-beta-D-glucopyranoside	A	-	-	-	-	-
75[ac]	Astragalin	A	B	A	★	=	☆
76[ac]	Cynaroside	A	B	A	★	=	☆
77[ac]	Kaempferol	A	B	A	★	=	☆
78[ac]	Scutellarein	A	B	A	★	=	☆
79[*]	Scutellarin	A	C	B	★	★	☆
80[bc]	Isorhamnetin	A	A	B	=	★	★
81[*]	Fisetin	C	A	B	☆	☆	★
82[bc]	Salicylic acid	-	-	A	-	-	-

No.	Compound Name	Comparison of median peak area					
		ys	ss	sx	ys-ss	ys-sx	ss-sx
83[a]	Azelaic acid	B	A	AB	☆	=	=
84*	Genistin	A	C	B	★	★	☆
85[ab]	Oroxin A	A	-	-	-	-	-
86*	Glabrolide	C	B	A	☆	☆	☆
87*	Phloretin	A	B	C	★	★	★
88[bc]	Linolenic acid ethyl ester	A	A	B	=	★	★
89*	Isopsoralen	C	B	A	☆	☆	☆
90*	Bergapten	C	B	A	☆	☆	☆
91*	Methoxsalen	C	B	A	☆	☆	☆
92*	Isobergapten	C	B	A	☆	☆	☆
93[ab]	Astringin	-	A	B	-	-	★
94*	Narirutin	B	A	C	☆	★	★
95	Norfenfluramine	A	A	A	=	=	=
96*	Pedunculoside	A	B	C	★	★	★
97*	Rosamultin	A	B	C	★	★	★
98*	Chrysin	A	B	-	★	-	-
99*	7,8-Dihydroxyflavone	A	B	-	★	-	-
100*	Rubiadin	A	B	C	★	★	★
101[ab]	6-Gingerol	B	A	A	☆	☆	=
102*	6-Shogaol	C	B	A	☆	☆	☆
103*	Medicagenic acid	A	B	C	★	★	★
104	Eplerenone	A	A	A	=	=	=
105*	Asiatic acid	A	B	C	★	★	★
106*	Ursolic acid	A	B	-	★	-	-
107*	Quillaic acid	B	A	C	☆	★	★
108[bc]	Tributyl Phosphate	A	A	B	=	★	★
109[ac]	Germacrone	-	A	-	-	-	-
110*	Aristolone	C	A	B	☆	☆	★
111*	alpha-Cyperone	C	A	B	☆	☆	★

No.	Compound Name	Comparison of median peak area					
		ys	ss	sx	ys-ss	ys-sx	ss-sx
112[ab]	(+)-Nootkatone	A	-	-	-	-	-
113[ab]	Methyl hexadecanoate	B	A	A	☆	☆	=
114[ac]	alpha-Linolenic acid	B	A	B	☆	=	★

Note: The two groups with no significant difference ($P \geq 0.05$) have been assimilated.
*: $P < 0.05$; a: There was comparison significance between ys and ss, $P < 0.05$; b: ys 与 sx There was comparison significance between ys and sx, $P < 0.05$; c: There was comparison significance between ss and sx, $P < 0.05$; ys: Wild Taihang Flower; ss: Artificially planting Taihang flowers on the mountain; sx: Artificial cultivation of taiwanese flowers under the mountain; all: A mixture of three batches of taiwanese flower extracts grown in different environments mixed in equal amounts. -: No.
Comparison of median peak areas of the three samples: the largest was A, the second was B, the third was C, and no difference with the remaining two samples was AB; ☆: Down-regulation; =: No difference; ★: Up-regulation

STable 3. The result of molecular docking

Compounds name	CID	S(Kcal/mol)	Amino acid residues(2QMJ)	Ligand/Receptor/Interaction
Potentillin	452242	-8.0015	VAL451, PHE450, GLU404, LYS480, THR204, ASP203, ARG526, THR205, ASP542, ASP443, MET444, PHE575, TRP406, TYR299, GLY602, ALA576, GLN603, TYR605	O12OD2/ASP542(A)/H-donor①, O13OD2/ASP203(A)/H-donor①, O22OE1/GLU404(A)/H-donor①
Ellagic Acid 4-O-Xylopyranoside	5487461	-7.6251	SER288, VAL779, LYS776, ALA285, HIS645, ALA780, ASP777, PRO287, SER521, THR775, ARG520, LEU286, PHE522, ILE523, ALA537, PHE535, ALA536	O3O/PHE535 (A)/H-donor, O4O/PHE535 (A)/H-donor, C16O/ARG520 (A)/H-donor, O3/N/ILE523(A)/ H-acceptor, O10N/ASP777 (A)/H-acceptor, O11CE/LYS534 (A)/H-acceptor①
Ellagic Acid Glucoside	91072206	-7.3979	ILE364, TRP406, TYR299, TRP441, ILE328, ASP327, PHE575, HIS600, TRP539, ASP443, MET444, ASP542, ARG526, SER448, ASP203, PHE450	O4OD2/ASP327(A)/H-donor①, O5OD1/ASP542(A)/H-donor①, O9OD2/ASP203(A)/H-donor①, C16OD2/ASP443(A)/H-donor①, O3NE2/HIS600(A)/H-acceptor①, O4NE2/HIS600(A)/H-acceptor①, O5NH1/ARG526(A)/H-acceptor①
Procyanidin B2-3'-O-gallate	15593124	-6.9372	TYR605, ASN207, THR211, LEU473, ASN209, THR205, ASP542, ASP203, THR204, ARG526, TYR214, MET444, ASP443, TRP406, PHE575, ALA576	O9OD2/ASP203 (A)/H-donor①, O10OD1/ASP203(A)/H-donor①, O11SD/MET444(A)/H-donor①, O13OD2/ASP542 (A)/H-donor①

**Compounds name**	**CID**	**S(Kcal/mol)**	**Amino acid residues(2QMJ)**	**Ligand/Receptor/Interaction**
4'-O-Arabinofuranosylellagic Acid	101129362	-6.6741	PHE450, TYR299, ASP327, ILE328, ILE364, TRP406, ASP443, ASP203, ARG526, ASP542, MET444, TRP539, PHE575, SER448, GLN603	O3OD2/ASP443(A)/H-donor①, O4OD1/ASP443(A)/H-donor①, O4OD2/ASP443(A)/H-donor①, O5OD1/ASP327(A)/H-donor①, O8OD2/ASP542(A)/H-donor①, O3NE1/TRP406(A)/H-acceptor①, O4NH1/ARG526(A)/H-acceptor①
Casuariin	14035442	-6.0355	LEU473, THR211, THR204, THR205, ASN209, ASN207, GLY208, THR544, THR546, ASP549, ASP548, TRP552	O14OD2/ASP549 (A)/H-donor①, O15OG1/THR544(A)/H-donor①, O13ND2/ASN207(A)/H-acceptor①, O18N/THR205(A)/H-acceptor
Ellagic Acid	5281855	-5.3858	ASP702, GLU704, LYS724, TYR626, ILE725, GLY726, LEU727, MET718, LEU720, HIS728, GLU719, TRP711	O3OE2/GLU704(A)/H-donor①, O8CA/GLY726(A)/H-acceptor
Phenylalanine	6140	-5.0462	LEU720, MET718, TYR626, ILE725, GLU719, LYS724, GLY726, GLU704, LEU727, TYR703, HIS728, ASP702	O1O/LEU727 (A)/H-donor, N3O/ILE725 (A)/H-donor, O2N/GLU704 (A)/H-acceptor, N3OH/TYR626 (A)/H-acceptor①

S: Binding energy; ①: sidechain.

STable 4. Cross-reference of active screening compounds (Table 2) with global metabolomics dataset (STable 1/STable 2)

No. (Table 2)	Compound name	RT (min) (Table 2)	Matched entry in STable 1	Peak area in sx (STable 2)	Detected globally?	Remarks
1	Proline	1.179	No. 4 (2-Pyrrolidinecarboxylic acid)	B	☑	Proline present as cyclized form; consistent.
2	HHDP-D-Glucose	1.625	–	–	✗	Related ellagitannins (No. 26, 55) present.
3	Phenylalanine	2.445	No. 17 (L-Phenylalanine)	A	☑	High abundance in sx; fully consistent.
4	Casuariin	3.143	–	–	✗	Large tannin; below Orbitrap detection limit.
5	Epicatechin	4.497	No. 30 (Epicatechin)	C	☑	Present in sx, lower abundance.
6	Tryptophan	4.525	No. 24 (L-Tryptophan)	A	☑	Present in sx; consistent.
7	Pedunculagin	4.818	–	–	✗	Hydrolyzable tannin; not annotated.
8	Chlorogenic acid	6.189	No. 33	A	☑	High abundance; fully consistent.
9	Catechin	6.501	–	–	✗	Isomer of epicatechin (No. 30); distinguished in Q-TOF.
10	BLicoagroside B	7.756	–	–	✗	Minor glycoside; only detected after targeted extraction.
11	Luteolin diglucuronide	9.568	–	–	✗	Low abundance flavone; not in global library.
12	Tricetin diglucuronide	11.284	–	–	✗	Similar to above.
13	Ellagic acid glucoside	12.384	– (No. 26, 55 related)	–	✗	Free glucoside not annotated; derivatives present.
14	Procyanidin B2-3′-O-gallate	14.196	–	–	✗	Large proanthocyanidin; not

No. (Table 2)	Compound name	RT (min) (Table 2)	Matched entry in STable 1	Peak area in sx (STable 2)	Detected globally?	Remarks
						annotated.
15	4′-O-Arabinofuranosylellagic acid	18.561	–	–	✕	Ellagic acid pentoside; below detection.
16	Ellagic acid 2-rhamnoside	18.690	–	–	✕	Not annotated.
17	Ellagic acid 4-O-xylopyranoside	19.809	–	–	✕	Not annotated.
18	Ellagic acid (free)	20.198	–	–	✕	Possibly suppressed in ESI- or co-eluted.
19	Potentillin	21.229	–	–	✕	High molecular weight tannin; optimized Q-TOF method detected it.
20	Isoquercitrin	21.932	No. 70	A	☑	Fully consistent.
21	Luteolin-7-O-glucoside	24.975	No. 76 (Cynaroside)	A	☑	Fully consistent (synonym).
22	Kaempferol-3-O-glucuronoside	25.052	– (No. 75 related)	–	⚠	Kaempferol glucoside (No. 75) present; glucuronoside not annotated.
23	Ducheside A	25.518	–	–	✕	Ellagic acid derivative; not annotated.

☑ = Detected and matched; ⚠ = Partially matched via related compound; ✕ = Not detected in global run.

Note: Among the 23 compounds listed in Table 2, 8 (proline, phenylalanine, epicatechin, tryptophan, chlorogenic acid, isoquercitrin, luteolin-7-O-glucoside, and indirectly kaempferol-3-O-glucuronoside) were positively identified in the global UPLC-MS/MS analysis of the mixed sample (STable 1), and their relative abundances in the sx extract are provided in STable 2. This confirms substantial chemical overlap between the two analytical datasets. The remaining 15 compounds are predominantly high-molecular-weight tannins (e.g., casuariin, pedunculagin, potentillin) and ellagic acid glycosides, which were either below the detection threshold of the Orbitrap run or poorly ionized under the original ESI conditions. Among these, seven of the eight α-glucosidase binders (Table 3) fall into this category – they were not detected in the global profiling but were successfully captured in the second analysis because the extract was prepared at a higher concentration, with longer ultrasonication, and analyzed by a Q-TOF platform optimized for tannin detection. Importantly, all 23 compounds originated from the same batch of sx plant material, and the 8 overlapping compounds demonstrate that the active screening was conducted on the same chemical matrix as the global profiling. The additional compounds identified in the second analysis represent a deeper, not contradictory, characterization of the sample.

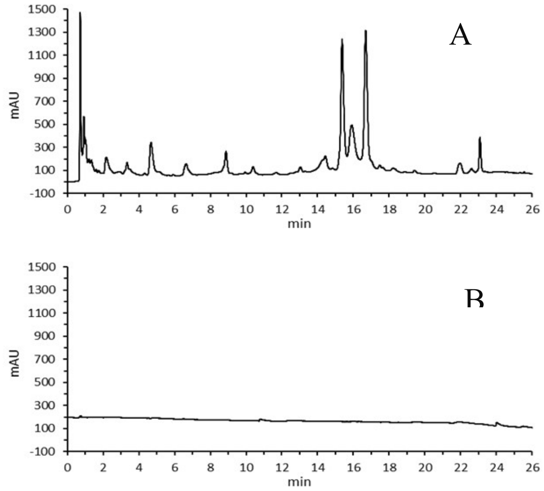

SFigure 1. Chromatograms of first online extraction (A) and second online extraction (B)

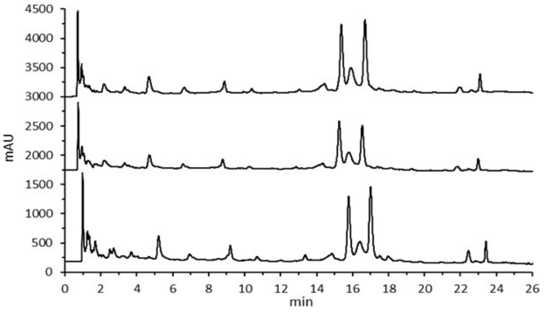

SFigure 2. Reproducibility chromatogram of online extraction sample

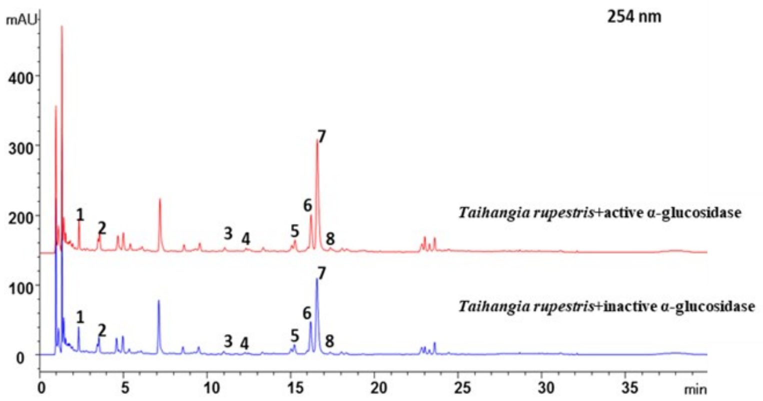

SFigure 3. Ultrafiltration centrifugal chromatogram of *T. rupestris*

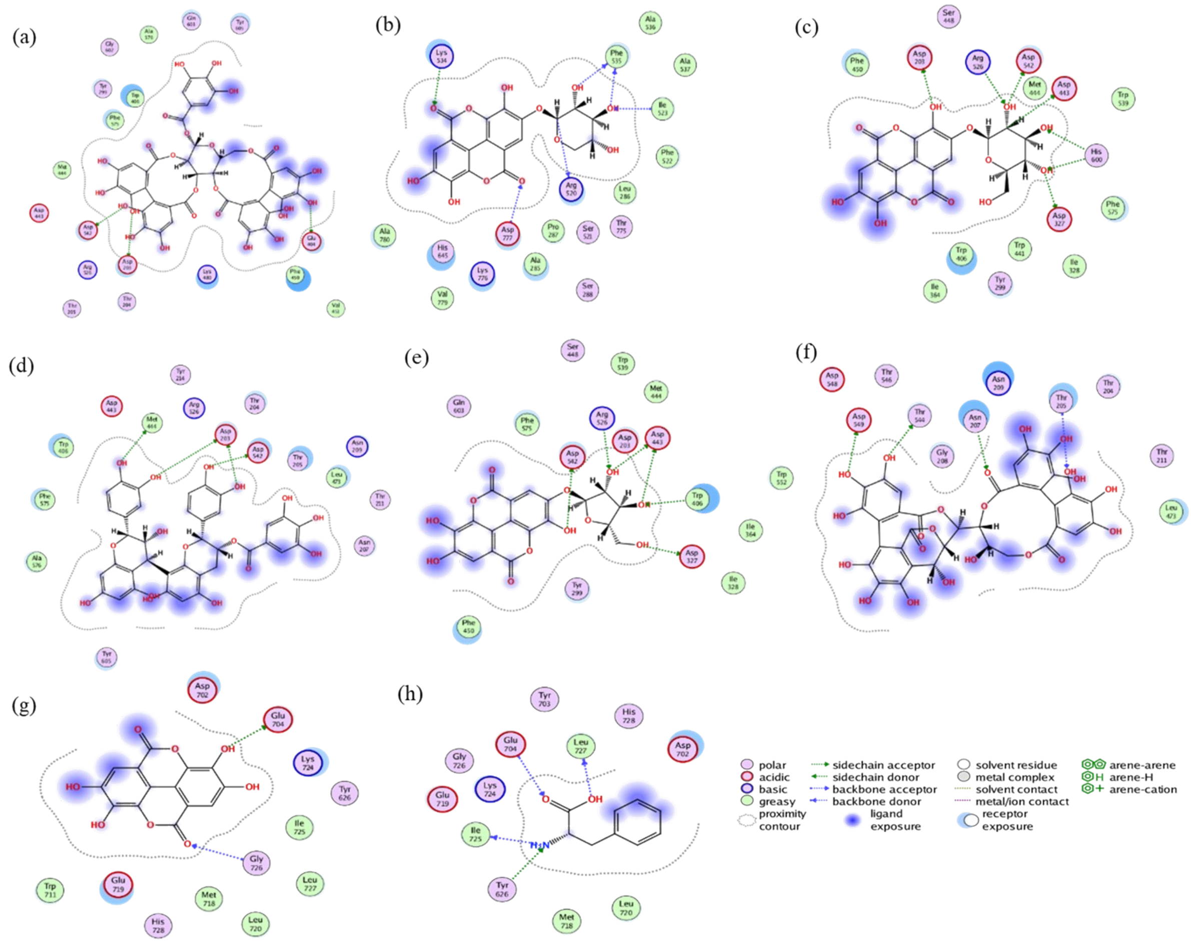

SFigure 4. Optimal conformation of the molecular docking between the active component of *T. rupestris* and α-glucosidase-associated protein (2QMJ) ((a): potentillin, (b): ellagic acid 4- o-xylopyranoside, (c): ellagic acid glucoside, (d): procyanidin B2-3'-o-gallate, (e): 4'-o-arabinofuranosylellagic acid, (f): casuariin, (g): ellagic acid, (h): phenylalanine)
